# Composition–structure–activity relationships in Cu_*x*_Co_1−*x*_Fe_2_O_4_ spinel ferrites for visible-light photo-Fenton degradation of rhodamine B

**DOI:** 10.1039/d6ra06896k

**Published:** 2026-09-30

**Authors:** Thi To Loan Nguyen, Thi Hien Lan Nguyen, Xuan Truong Mai, Thi Hau Vu, Thi Mai Viet Ngo, Van Nhuong Vu, Truong Xuan Vuong

**Affiliations:** a Faculty of Chemistry, Thai Nguyen University of Education (TNUE) No. 20 Luong Ngoc Quyen Street Thai Nguyen City 24000 Vietnam; b Faculty of Natural Sciences and Technology, TNU-University of Science Tan Thinh Ward Thai Nguyen City 24000 Vietnam xuanvt@tnus.edu.vn; c Applied Chemistry Research Group, Thai Nguyen University of Education (TNUE) No. 20 Luong Ngoc Quyen Street Thai Nguyen City 24000 Vietnam

## Abstract

Although Cu-substituted CoFe_2_O_4_ ferrites have been extensively investigated as visible-light photo-Fenton catalysts, the physicochemical basis of their composition-dependent activity maximum remains insufficiently resolved. Herein, a systematic Cu_*x*_Co_1−*x*_Fe_2_O_4_ series (*x* = 0–0.5) was synthesized within a single spinel-ferrite framework, enabling crystallographic, vibrational, morphological, textural, optical, and catalytic characteristics to be evaluated consistently across the investigated composition range. The cubic spinel structure was retained throughout the series, while Cu substitution induced systematic changes in lattice characteristics, local coordination-related vibrational features, microstructure, and optical properties. These changes were most pronounced at intermediate Cu contents (*x* = 0.3–0.4), whereas the optical band-gap energies remained within 1.34–1.60 eV. Rhodamine B degradation increased from 76.45% for pristine CoFe_2_O_4_ to 98.78% for Cu_0_._4_Co_0_._6_Fe_2_O_4_, accompanied by an approximately 2.9-fold increase in the apparent reaction-rate constant. The optimized catalyst retained approximately 92% of its initial degradation efficiency after four consecutive reuse cycles. Mineralization assessment further indicated substantial, although incomplete, organic-carbon removal, with 84.56% TOC removal at 240 min and 90.8% COD removal at 360 min under the investigated conditions. Radical-scavenging experiments supported ˙OH as the predominant reactive species, while the elementary radical-generation pathways remained unresolved. A central finding is that the highest photocatalytic activity did not coincide with either the maximum BET surface area or the minimum optical band-gap energy. The activity maximum occurred alongside pronounced changes in crystallographic and local coordination-related vibrational characteristics, but these features cannot be assigned as independent causal determinants from the present dataset. Within the limits of the present dataset, the activity trend is therefore more consistently associated with the coupled evolution of composition-dependent physicochemical characteristics than with any single measured descriptor. These findings provide experimentally supported insight into the composition–structure–activity relationships observed within the investigated Cu_*x*_Co_1−*x*_Fe_2_O_4_ system.

## Abstract 2

Although Cu-substituted CoFe_2_O_4_ ferrites have been extensively investigated as visible-light photo-Fenton catalysts, the physicochemical basis of their composition-dependent activity maximum remains insufficiently resolved.

## Graphical abstract

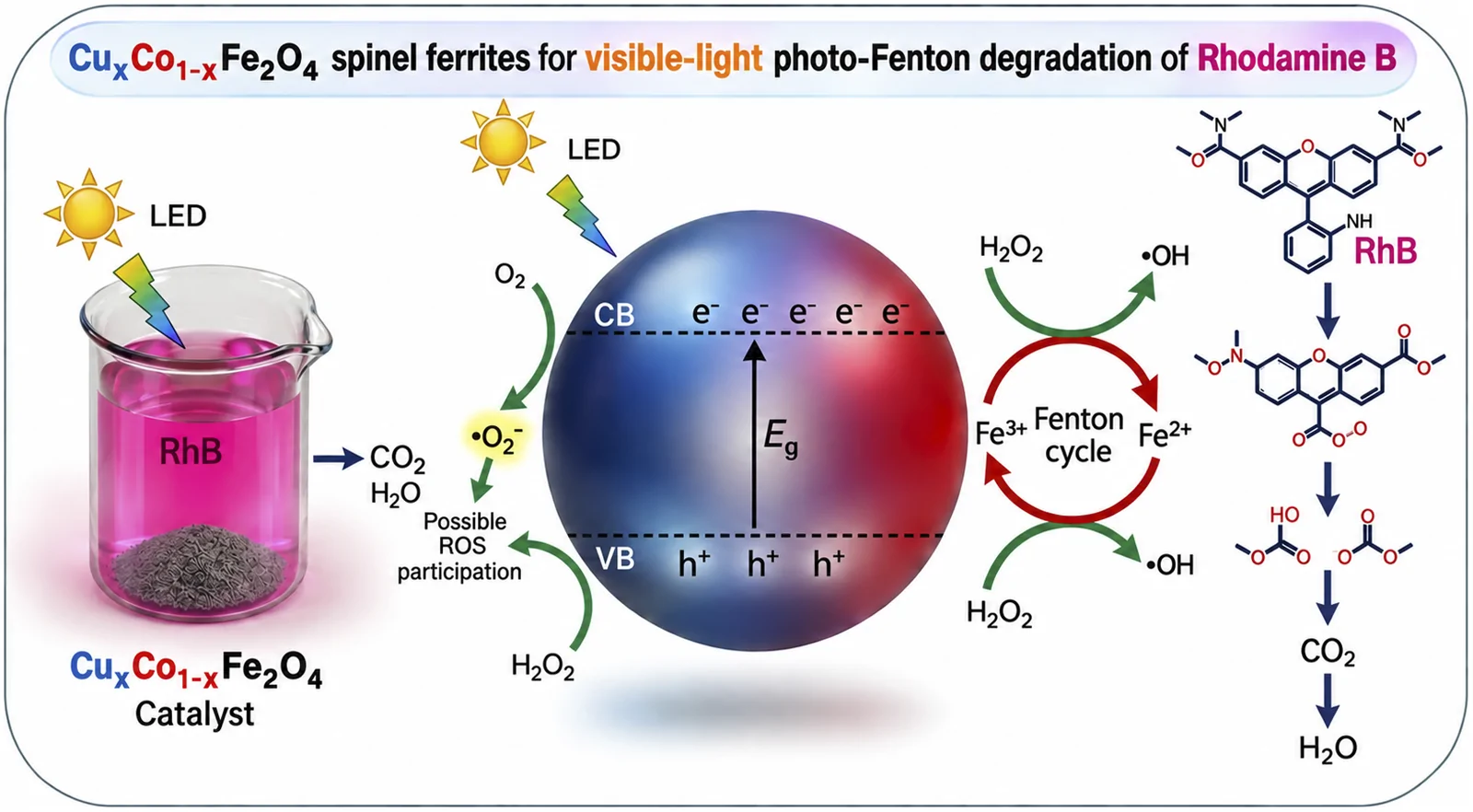

## 1. Introduction

The rapid expansion of textile, printing, paper, leather, cosmetic, and dye-manufacturing industries has substantially increased the discharge of dye-containing wastewater into natural aquatic environments. Unlike many conventional organic contaminants, synthetic dyes are deliberately designed to resist chemical, thermal, and photochemical degradation during industrial processing, allowing considerable quantities to persist in aquatic systems when treatment is incomplete.^1,2^ Even at relatively low concentrations, dissolved dyes reduce light penetration, suppress photosynthetic activity, and disrupt aquatic ecosystems.^3,4^ Among these pollutants, Rhodamine B (RhB) is one of the most widely employed model contaminants because of its extensive industrial use, intense coloration, high molar absorptivity, and exceptional resistance to natural degradation. Toxicological investigations have further associated RhB exposure with mutagenic, carcinogenic, and neurotoxic effects, reinforcing the need for efficient remediation technologies.^5–8^

A wide range of physicochemical and biological treatment technologies, including adsorption, coagulation–flocculation, membrane filtration, and biological processes, has been developed for dye removal.^3,4^ However, each approach remains constrained by intrinsic limitations. Adsorption transfers pollutants from the liquid phase to a solid adsorbent requiring subsequent regeneration or disposal, membrane processes are susceptible to fouling and elevated operating costs, whereas biological treatments generally exhibit limited effectiveness toward chemically stable aromatic dyes.^6^ As a consequence, incomplete mineralization and secondary waste generation continue to challenge conventional treatment technologies. Advanced oxidation processes (AOPs), which generate highly reactive oxidative species *in situ*, provide an attractive alternative for degrading refractory organic contaminants into less harmful products.^9,10^ Among these technologies, heterogeneous visible-light photo-Fenton systems have attracted considerable attention because they couple hydrogen peroxide activation with light-assisted catalysis while avoiding many of the operational drawbacks of conventional homogeneous Fenton chemistry. Under suitable reaction conditions, hydroxyl radicals (˙OH) generated on the catalyst surface participate in the oxidative degradation of persistent organic pollutants. Compared with homogeneous Fenton systems, heterogeneous catalysts facilitate catalyst recovery, suppress iron-sludge generation, and generally exhibit superior operational stability.^11–13^ Their ability to utilize visible light, which accounts for the largest proportion of the solar spectrum reaching the Earth's surface, further enhances their attractiveness for sustainable wastewater treatment.^12,13^

Among the catalyst families investigated for heterogeneous visible-light photo-Fenton processes, spinel ferrites have attracted particular interest because they combine structural flexibility, chemical stability, magnetic separability, and favorable redox characteristics within a single crystalline framework.^14^ Spinel ferrites, with the general formula MFe_2_O_4_, readily accommodate gradual cation substitution while preserving the fundamental spinel lattice, providing an ideal platform for systematically examining how compositional variation influences crystallographic characteristics, local coordination, physicochemical properties, and catalytic performance.^14,15^ Within this family, CoFe_2_O_4_ has been widely investigated owing to its chemical robustness, strong magnetic response, and efficient interaction with hydrogen peroxide under heterogeneous photo-Fenton conditions.^15–18^ Previous studies have shown that modifying the cation composition of CoFe_2_O_4_ alters multiple measurable physicochemical characteristics simultaneously, including crystal structure, local coordination environments, morphology, surface properties, optical response, and catalytic activity.^14,17^ Consequently, CoFe_2_O_4_-based spinel ferrites provide an appropriate model system for investigating composition–structure–activity relationships in heterogeneous photo-Fenton catalysis.

Among the transition-metal substitutions explored for CoFe_2_O_4_, Cu incorporation is particularly attractive because Cu^2+^ possesses ionic characteristics that differ appreciably from those of Co^2+^ while remaining structurally compatible with the spinel lattice. Previous studies have shown that gradual Cu substitution modifies lattice parameters, crystallographic refinement, local metal–oxygen coordination, microstructure, optical absorption, magnetic behavior, and catalytic performance.^19,20^ Consequently, Cu-substituted CoFe_2_O_4_ ferrites offer a well-controlled composition series for examining how progressive compositional modification influences the coordinated evolution of measurable physicochemical characteristics within a common spinel framework. Rather than introducing additional interfaces or secondary phases, this approach isolates composition as the primary experimental variable, thereby enabling intrinsic composition-dependent effects to be evaluated more systematically.

Consistent with these observations, numerous studies have explored Cu-substituted CoFe_2_O_4_ ferrites for photocatalytic and visible-light photo-Fenton degradation of rhodamine B and other organic pollutants, frequently reporting composition-dependent variations in catalytic performance together with concurrent changes in crystal structure, optical response, magnetic properties, and surface characteristics.^20–22^ Despite these advances, the physicochemical origin of the optimum catalyst composition remains unresolved. Catalytic enhancement has alternatively been associated with crystallite size, optical band-gap energy, surface area, lattice distortion, or Cu content itself. Because these physicochemical characteristics evolve concurrently during progressive cation substitution, studies focusing primarily on individual descriptors or selected catalyst compositions have not fully resolved how their coordinated evolution influences catalytic performance across an entire composition series. As a result, systematic experimental evidence linking composition-dependent physicochemical evolution to catalytic activity within a single spinel framework remains limited, hindering a unified interpretation of composition-dependent behavior in Cu-substituted spinel ferrites.

Accordingly, the central scientific question is no longer whether Cu substitution enhances the visible-light photo-Fenton performance of CoFe_2_O_4_, but rather why the highest catalytic activity consistently emerges only within a limited compositional interval despite the continuous evolution of crystallographic and physicochemical characteristics. Addressing this question requires more than comparing the catalytic performance of individual compositions. Instead, it requires systematic evaluation of how crystallographic, vibrational, morphological, textural, optical, and catalytic characteristics evolve concurrently across an entire composition series under identical synthesis and reaction conditions. Such an experimental framework enables catalytic performance to be interpreted from the perspective of composition–structure–activity relationships, rather than attributing activity changes to any single physicochemical descriptor considered in isolation.

To address this question, the present study systematically integrates crystallographic, vibrational, morphological, textural, optical, kinetic, and catalytic analyses across a complete Cu_*x*_Co_1−*x*_Fe_2_O_4_ (*x* = 0–0.5) composition series synthesized under identical experimental conditions.^23,24^ Rather than comparing the performance of selected catalyst compositions or interpreting activity through individual physicochemical descriptors, the present work evaluates the coordinated evolution of measurable physicochemical characteristics across the entire substitution range. This integrated experimental framework provides an experimentally supported basis for understanding how composition-induced physicochemical evolution is associated with catalytic performance and for interpreting composition–structure–activity relationships in Cu-substituted spinel ferrites under visible-light photo-Fenton conditions.

## 2. Materials and methods

### 2.1. Chemicals

Iron(iii) nitrate nonahydrate (Fe(NO_3_)_3_·9H_2_O, China), cobalt(ii) nitrate hexahydrate (Co(NO_3_)_2_·6H_2_O, Merck), copper(ii) nitrate trihydrate (Cu(NO_3_)_2_·3H_2_O, Merck), and urea (CO(NH_2_)_2_, Merck) were used for ferrite synthesis. Rhodamine B (RhB, C_28_H_31_ClN_2_O_3_, Merck) was employed as the model organic pollutant. Hydrogen peroxide (H_2_O_2_, 30 wt%, density 1.11 g cm^−3^, China) served as the oxidizing agent during photo-Fenton experiments. Deionized water was used throughout all synthesis and catalytic procedures.

All chemicals were of analytical grade and were used as received without further purification.

### 2.2. Synthesis of Cu_*x*_Co_1−*x*_Fe_2_O_4_ spinel ferrites

#### 2.2.1. Preparation of precursor solutions

A series of Cu-substituted cobalt ferrites with nominal compositions Cu_*x*_Co_1−*x*_Fe_2_O_4_ (*x* = 0–0.5) was synthesized *via* the gel combustion method using a nitrate–urea precursor system. Stoichiometric amounts of Fe(NO_3_)_3_·9H_2_O, Co(NO_3_)_2_·6H_2_O, Cu(NO_3_)_2_·3H_2_O, and urea were weighed according to the compositions summarized in Table 1. The metal salts were dissolved in deionized water under continuous magnetic stirring until homogeneous precursor solutions were obtained. To maintain consistent synthesis conditions across the entire composition series, the amount of urea was kept constant for all samples.

**Table 1:** Nominal compositions and precursor molar ratios used for Cu_*x*_Co_1−*x*_Fe_2_O_4_ synthesis

Sample	Nominal composition	Urea (mol)	Fe(NO_3_)_3_ (mol)	Co(NO_3_)_2_ (mol)	Cu(NO_3_)_2_ (mol)
CFO	CoFe_2_O_4_	0.075	0.020	0.010	0
Cu_0.1_-CFO	Cu_0_._1_Co_0_._9_Fe_2_O_4_	0.075	0.020	9 × 10^−3^	1 × 10^−3^
Cu_0.2_-CFO	Cu_0_._2_Co_0_._8_Fe_2_O_4_	0.075	0.020	8 × 10^−3^	2 × 10^−3^
Cu_0.3_-CFO	Cu_0_._3_Co_0_._7_Fe_2_O_4_	0.075	0.020	7 × 10^−3^	3 × 10^−3^
Cu_0.4_-CFO	Cu_0_._4_Co_0_._6_Fe_2_O_4_	0.075	0.020	6 × 10^−3^	4 × 10^−3^
Cu_0.5_-CFO	Cu_0_._5_Co_0_._5_Fe_2_O_4_	0.075	0.020	5 × 10^−3^	5 × 10^−3^

#### 2.2.2. Preparation of ferrite powders

The precursor solutions were heated at 70 °C under continuous stirring. Progressive evaporation of water increased solution viscosity and eventually resulted in the formation of homogeneous gels. The gels were subsequently maintained at 70 °C for approximately 72 h to remove residual moisture and obtain dry precursor solids.

The dried precursor was subsequently calcined in air at 600 °C for 3 h. Upon heating, the nitrate–urea precursor underwent an exothermic redox combustion process, with the metal nitrates acting as oxidizing components and urea serving as the fuel. Thermal decomposition of the nitrate precursors resulted in the formation of Cu-, Co-, and Fe-containing oxide species within the intimately mixed precursor. Subsequent thermal treatment promoted their interaction and crystallization into the Cu_*x*_Co_1−*x*_Fe_2_O_4_ spinel ferrite phase.

Identical thermal-treatment conditions were applied to all compositions to ensure consistency across the composition series. A schematic illustration of the synthesis procedure is shown in Fig. 1.

**Fig. 1:**
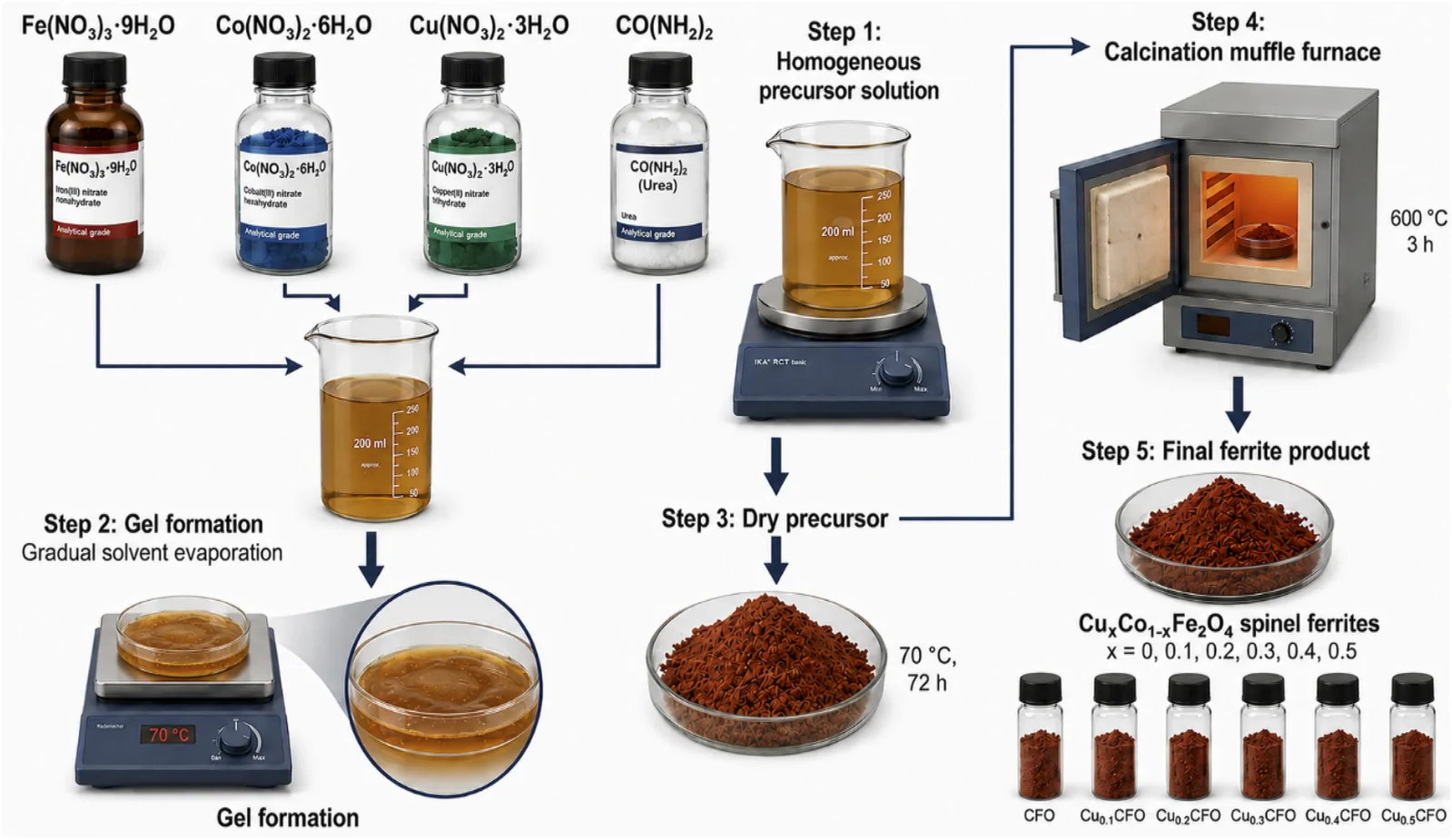
Schematic illustration of the synthesis procedure for Cu_*x*_Co_1−*x*_Fe_2_O_4_ (*x* = 0–0.5) spinel ferrites *via* the gel combustion method.

The synthesized ferrites were designated according to their nominal Cu-substitution levels as CFO, Cu_0.1_-CFO, Cu_0.2_-CFO, Cu_0.3_-CFO, Cu_0.4_-CFO, and Cu_0.5_-CFO.

### 2.3. Material characterization

#### 2.3.1. X-ray diffraction (XRD)

The phase composition and crystal structure of the synthesized ferrites were investigated using a Bruker D2 Advance X-ray diffractometer equipped with Cu Kα radiation (*λ* = 0.154056 nm). Diffraction patterns were recorded over the 2*θ* range of 20–70° with a step size of 0.03° at an operating voltage of 30 kV and a tube current of 30 mA. Phase identification was performed by comparison with standard diffraction data for spinel ferrites.

The average crystallite size (*D*) was estimated using the Scherrer equation based on the full width at half maximum (FWHM) of the most intense (311) diffraction reflection after correction for instrumental broadening:

1 *D* = *Kλ*/(*β* cos *θ*)

where *K* is the shape factor (0.9), *λ* is the X-ray wavelength, *β* is the corrected FWHM of the diffraction peak, and *θ* is the corresponding Bragg angle.

#### 2.3.2. Fourier-transform infrared spectroscopy (FTIR)

Fourier-transform infrared (FTIR) spectra were recorded using an FTIR Affinity-1S spectrometer (Shimadzu, Japan) employing the KBr pellet technique. Spectra were collected in the range of 4000–400 cm^−1^ with a spectral resolution of 4 cm^−1^. The obtained spectra were used to identify characteristic vibrational bands associated with the spinel ferrite structure.

#### 2.3.3. Scanning electron microscopy (SEM)

The surface morphology of the synthesized ferrites was examined using a JEOL-5300 scanning electron microscope (Japan) operated at an accelerating voltage of 15 kV. SEM images were employed to evaluate particle morphology, surface texture, and agglomeration characteristics of the prepared materials.

#### 2.3.4. Transmission electron microscopy (TEM)

Microstructural features were further investigated using a JEOL JEM-1010 transmission electron microscope (Japan) operated at an accelerating voltage of 100 kV. TEM observations were performed to determine particle morphology and particle-size characteristics at the nanoscale.

#### 2.3.5. Energy-dispersive X-ray spectroscopy (EDX)

Elemental composition was analyzed using an energy-dispersive X-ray spectroscopy (EDX) detector attached to a Hitachi S-4800 field-emission scanning electron microscope (Japan). EDX spectra were used to determine the elemental composition and verify the presence of Co, Cu, Fe, and O in the synthesized ferrites. Semi-quantitative elemental analyses were obtained from the corresponding EDX spectra.

#### 2.3.6. Nitrogen adsorption–desorption analysis

Textural properties were determined from nitrogen adsorption–desorption measurements using a Tristar 3000 Micromeritics surface-area analyzer (USA). Prior to analysis, the samples were degassed under vacuum at 150 °C for 12 h to remove physically adsorbed moisture and impurities. Adsorption–desorption isotherms were recorded at −196 °C using liquid nitrogen.

The specific surface area (SBET) was calculated using the Brunauer–Emmett–Teller (BET) method. Total pore volume was determined from the amount of nitrogen adsorbed at a relative pressure close to unity, while average pore diameter and pore-size distribution were estimated using the Barrett–Joyner–Halenda (BJH) method.

#### 2.3.7. UV-vis diffuse reflectance spectroscopy (DRS)

Optical absorption properties were investigated using a Cary 5000 UV-vis-NIR spectrophotometer (Agilent Technologies) operated in diffuse-reflectance mode. Diffuse reflectance spectra were collected over the wavelength range of 200–800 nm and converted into Kubelka–Munk functions according to:

2 *F*(*R*) = (1 − *R*)^2^/(2*R*)

where *F*(*R*) is the Kubelka–Munk function, expressed as *F*(*R*) = (1 − *R*)^2^/(2*R*), and *R* is the measured diffuse reflectance. The optical band-gap energy (*E*_g_) was estimated using the Wood–Tauc method based on plots of [*F*(*R*)*hν*]^*n*^*versus* photon energy (*hν*), where *n* = 2 was adopted assuming a direct allowed electronic transition. Band-gap values were determined by extrapolating the linear portion of the corresponding Wood–Tauc plots to the photon-energy axis.

### 2.4. Visible-light photo-Fenton experiments

#### 2.4.1. Rhodamine B calibration and adsorption-equilibrium determination

A calibration curve for Rhodamine B (RhB) was established using a series of standard solutions with concentrations ranging from 1 to 10 mg L^−1^ at pH 7. The absorbance values were measured at the maximum absorption wavelength (*λ*_max_ = 553 nm) using a UV-vis spectrophotometer. Linear regression analysis yielded the following calibration equation:

3 *A* = 0.165*C* + 0.0202

with a coefficient of determination (*R*^2^) of 0.99, where *A* is the absorbance and *C* is the RhB concentration (mg L^−1^). The small positive regression intercept reflects the empirical fit to the calibration data and should not be interpreted as the absorbance of distilled water at zero RhB concentration. The corresponding calibration data and UV-vis spectra are presented in Table S1 and Fig. S1 of the SI.

For samples yielding absorbance values within the established linear calibration range, no dilution was required prior to UV-vis measurement. Samples yielding absorbance values outside this range were appropriately diluted before measurement, and the corresponding dilution factors were applied to calculate the RhB concentrations in the original samples. For the 15 mg L^−1^ initial-concentration experiment, early-stage samples were diluted twofold when necessary to maintain the measured absorbance within the established linear range.

Prior to the photocatalytic experiments, the dark adsorption behaviour of RhB was evaluated using CoFe_2_O_4_ to determine an appropriate pre-irradiation equilibration time. Briefly, 0.10 g of catalyst was dispersed in 100 mL of RhB solution (10 mg L^−1^, pH 7) in a 250 mL Erlenmeyer flask and magnetically stirred at 500 rpm in the dark at 25 ± 2 °C. Aliquots were withdrawn at regular intervals to monitor RhB adsorption as a function of contact time. The residual RhB concentration at each contact time was determined from the calibration equation (eqn (3)). The dark-adsorption removal percentage was subsequently calculated using eqn (4), where *C*_0_ represents the initial RhB concentration before contact with the catalyst and *C*_*t*_ represents the residual RhB concentration after dark adsorption for time *t*.

#### 2.4.2. Effect of catalyst composition

The influence of Cu substitution on visible-light-assisted photo-Fenton activity was investigated using CFO, Cu_0.1_-CFO, Cu_0.2_-CFO, Cu_0.3_-CFO, Cu_0.4_-CFO, and Cu_0.5_-CFO.

For each experiment, 100 mL of RhB solution (10 mg L^−1^, pH 7) was mixed with 0.10 g of catalyst in a 250 mL Erlenmeyer flask. The suspension was magnetically stirred at 500 rpm in the dark for 30 min to establish a stable adsorption baseline. After this equilibration period, a 5 mL aliquot was withdrawn, centrifuged, and analyzed at 553 nm to determine the RhB concentration used as *C*_0_. For kinetic comparison, the 30 min period was treated as sufficient to minimize further adsorption during subsequent irradiation under the investigated conditions.

Subsequently, 1.0 mL of 30 wt% H_2_O_2_ solution was added as the oxidant. The solution had a density of 1.11 g mL^−1^; thus, 1.0 mL contained 0.333 g H_2_O_2_ (9.79 mmol), corresponding to an initial H_2_O_2_ concentration of 97.9 mM in a total reaction volume of 100 mL. Visible-light irradiation was then initiated using a 30 W white LED lamp (emission range: 400–800 nm) positioned approximately 20 cm above the solution surface. The light intensity at the reactor surface was approximately 45 mW cm^−2^. The reaction mixture was continuously stirred at 500 rpm and maintained at 25 ± 2 °C throughout the experiment. Irradiation was continued for 240 min.

At 30 min intervals, 5 mL aliquots were withdrawn and immediately centrifuged to separate catalyst particles. The resulting supernatant was analyzed immediately to minimize post-sampling reactions. When necessary, the pH of the supernatant was adjusted to pH 7 using dilute HCl or NaOH solutions prior to UV-vis analysis. Residual RhB concentrations (*C*_*t*_) were determined from the calibration equation described above.

For comparison, control experiments involving H_2_O_2_/light and catalyst/light systems were conducted under otherwise identical conditions.

#### 2.4.3. Effect of H_2_O_2_ dosage

The influence of oxidant dosage was evaluated using Cu_0.4_-CFO as the catalyst.

Each experiment was performed using 100 mL of RhB solution (10 mg L^−1^, pH 7) and 0.10 g of catalyst. Following dark equilibration for 30 min, H_2_O_2_ (30 wt%) was introduced at dosages of 1.0, 1.5, and 2.0 mL, corresponding to approximately 98, 147 and 196 mM, respectively.

The suspensions were irradiated under visible light for 180 min while being continuously stirred at 500 rpm.

Aliquots were collected every 30 min, centrifuged, and analyzed according to the procedure described above. Degradation efficiencies were calculated using eqn (4).

4 RhB degradation (%) = [(*C*_0_ − *C*_*t*_)/*C*_0_] × 100

where *C*_0_ and *C*_*t*_ denote the RhB concentrations (mg L^−1^) before irradiation and after irradiation for time *t*, respectively.

#### 2.4.4. Effect of catalyst dosage

The influence of catalyst loading was examined using Cu_0.4_-CFO dosages of 0.075, 0.10, and 0.15 g. The experiments were conducted in 100 mL of RhB solution (10 mg L^−1^, pH 7) containing 1.5 mL of H_2_O_2_ (30 wt%, approximately 150 mM). After adsorption equilibrium had been established in the dark for 30 min, visible-light irradiation was applied for 180 min under continuous stirring at 500 rpm. Samples were withdrawn every 30 min and analyzed following the procedure described in Section 2.4.2.

#### 2.4.5. Effect of initial rhodamine B concentration

The effect of pollutant concentration was investigated using RhB solutions with initial concentrations of 5, 10, and 15 mg L^−1^.

For each experiment, 100 mL of RhB solution was mixed with 0.10 g of Cu_0.4_-CFO and stirred at 500 rpm in the dark for 30 min. Subsequently, 1.5 mL of H_2_O_2_ (30 wt%, approximately 150 mM) was added, and the suspension was irradiated under visible light for 180 min at 25 ± 2 °C.

Aliquots were withdrawn every 30 min, centrifuged immediately, and analyzed at 553 nm to determine the residual RhB concentration.

#### 2.4.6. Chemical oxygen demand (COD) analysis

Mineralization was assessed by monitoring the chemical oxygen demand (COD) of real textile wastewater collected from the wastewater storage pond of the An Le traditional mat-dyeing village, Dong Bang Commune, Hung Yen Province, Vietnam. Owing to its high color intensity and organic content, the wastewater was diluted 20-fold with deionized water prior to treatment.

Photo-Fenton experiments were carried out using 200 mL of the diluted wastewater in a 500 mL Erlenmeyer flask. The initial pH was adjusted to 7.0 before adding 0.10 g of Cu_0_._4_Co_0_._6_Fe_2_O_4_ catalyst. After 30 min of dark stirring to establish adsorption–desorption equilibrium, H_2_O_2_ (0.20 M), corresponding to the optimum condition determined in the optimization experiments, was introduced, and the suspension was irradiated with a 30 W visible-light LED lamp at room temperature.

At selected irradiation intervals, 5 mL aliquots were withdrawn and centrifuged to remove the catalyst. Residual H_2_O_2_ was eliminated by treating 2.5 mL of the supernatant with two drops of 20 wt% NaOH followed by heating at 100 °C for 15 min. A further 2.5 mL aliquot was mixed with 1.5 mL of digestion reagent and 3.5 mL of acidic dichromate reagent, digested at 150 °C for 2 h, cooled to room temperature, and analyzed spectrophotometrically at 616 nm. COD concentrations were determined from a calibration curve using the dichromate colorimetric method based on the standard dichromate digestion procedure.

The COD removal efficiency was calculated according to eqn (5):

5 COD removal (%) = [(COD_0_ − COD_*t*_)/COD_0_] × 100

where COD_0_ and COD_*t*_ are the chemical oxygen demand (mg L^−1^) of the solution before treatment and after irradiation for time *t*, respectively. All measurements were performed in triplicate, and the results are reported as the mean ± standard deviation.

#### 2.4.7. Radical-scavenging experiments

Reactive species involved in the visible-light photo-Fenton degradation of RhB were investigated by scavenger experiments using the optimum Cu_0_._4_Co_0_._6_Fe_2_O_4_ catalyst. Benzoquinone (BQ, 1 mM), isopropyl alcohol (IPA, 1 mM), and ethylenediaminetetraacetic acid (EDTA, 1 mM) were employed as scavengers for superoxide radicals (˙O_2_^−^), hydroxyl radicals (˙OH), and photogenerated holes (h^+^), respectively.

The experiments were performed using 100 mL of RhB solution (10 mg L^−1^, pH 7.0) containing 0.10 g of catalyst (1.0 g L^−1^) and H_2_O_2_ (0.15 M), corresponding to the optimum operating conditions determined in the preceding optimization experiments. The appropriate scavenger was added before visible-light irradiation, and the suspension was stirred in the dark for 30 min to establish adsorption–desorption equilibrium. The reaction mixture was then irradiated under a 30 W visible-light LED lamp with continuous magnetic stirring. Aliquots were collected at predetermined time intervals, centrifuged to remove the catalyst, and the residual RhB concentration was determined spectrophotometrically as described in Sections 2.4.1 and 2.4.2. The degradation efficiency was calculated according to eqn (4). All experiments were performed in triplicate, and the results are reported as the mean ± standard deviation.

#### 2.4.8. Reusability experiments

The reusability of the Cu_0_._4_Co_0_._6_Fe_2_O_4_ catalyst was assessed over four consecutive photo-Fenton degradation cycles. After each reaction, the catalyst was recovered by magnetic separation followed by centrifugation, washed thoroughly with deionized water and ethanol, and dried at 70 °C before being reused in the subsequent cycle.

For each recycling run, fresh RhB solution and H_2_O_2_ were used, whereas the catalyst dosage, initial RhB concentration, irradiation conditions, reaction duration, and sampling procedure were identical to those described in Sections 2.4.1–2.4.5. Residual RhB concentrations were measured spectrophotometrically as described in Section 2.4.1, and the degradation efficiency was calculated according to eqn (4). All recycling experiments were performed in triplicate, and the results are reported as the mean ± standard deviation.

### 2.5. Degradation efficiency and kinetic analysis

The degradation efficiency of RhB was calculated using eqn (4), where *C*_0_ is the RhB concentration after adsorption equilibrium and before visible-light irradiation, and *C*_*t*_ is the RhB concentration at irradiation time *t*.

The degradation kinetics were evaluated using a pseudo-first-order model:

6 ln(*C*_0_/*C*_*t*_) = *kt*

where *k* is the apparent pseudo-first-order rate constant (min^−1^) and *t* is the irradiation time (min).

The value of *k* was obtained from the slope of the linear regression of ln(*C*_0_/*C*_*t*_) *versus* irradiation time, while the corresponding coefficient of determination (*R*^2^) was used to assess the goodness of fit. The pseudo-first-order model was adopted as an empirical approach for comparing the relative degradation rates of the investigated ferrite catalysts, rather than as a mechanistic description of the underlying photo-Fenton reaction. This analysis assumes that the concentration-dependent degradation behaviour can be reasonably approximated by a pseudo-first-order relationship over the investigated irradiation interval. Because minor deviations from linearity were observed at longer irradiation times, particularly as RhB degradation approached completion, the resulting rate constants are therefore interpreted as apparent comparative kinetic parameters rather than intrinsic mechanistic constants.

### 2.6. Data processing

Graphical plotting, linear-regression analysis, and kinetic fitting were performed using Origin Pro 2019 software and R (ggplot2). Quantitative degradation and reusability results were obtained from triplicate experiments and are reported as mean ± standard deviation to account for experimental variability.

## 3. Results and discussion

### 3.1. Characterization of studied materials

#### 3.1.1. XRD analysis

The X-ray diffraction patterns of pristine CoFe_2_O_4_ (CFO) and Cu-substituted Cu_*x*_Co_1−*x*_Fe_2_O_4_ ferrites (*x* = 0–0.5), together with the crystallographic parameters derived from XRD, are presented in Fig. 2 and Table 2.

**Fig. 2:**
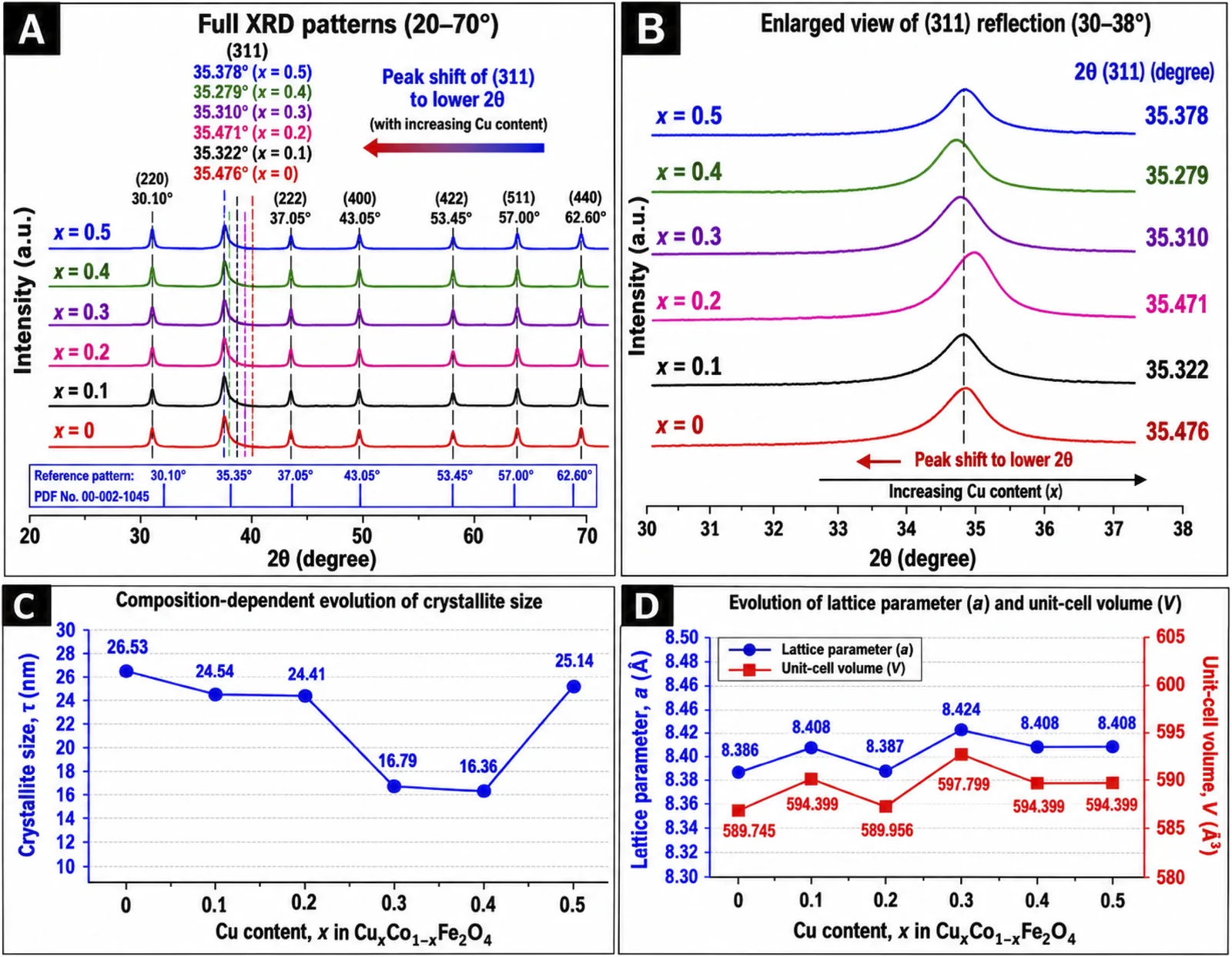
X-ray diffraction (XRD) analysis of Cu_*x*_Co_1−*x*_Fe_2_O_4_ (*x* = 0–0.5) spinel ferrites. (A) Full XRD patterns in the 2*θ* range of 20–70°. (B) Enlarged view of the (311) reflection showing the composition-dependent peak shift. (C) Crystallite size estimated from the Scherrer equation as a function of Cu substitution. (D) Variation of the lattice parameter (*a*) and unit-cell volume (*V*) with Cu content.

**Table 2:** Crystallographic parameters of pristine CoFe_2_O_4_ and Cu-substituted Cu_*x*_Co_1−*x*_Fe_2_O_4_ ferrites derived from X-ray diffraction analysis. The table summarizes the position of the (311) reflection (2*θ*_311_), interplanar spacing (*d*_311_), full width at half maximum (FWHM), Scherrer crystallite size (*D*), dislocation density (*δ*), lattice parameter (*a*), and unit-cell volume (*V*)

Sample	2*θ*_311_ (°)	*d*_311_ (Å)	FWHM (°)	*D* (nm)	*δ* (×10^−3^ nm^−2^)	*a* (Å)	*V* (Å^3^)
CoFe_2_O_4_	35.476	2.5316	0.311	26.53	1.421	8.386	589.745
Cu_0_._1_Co_0_._9_Fe_2_O_4_	35.322	2.5422	0.336	24.54	1.661	8.408	594.399
Cu_0_._2_Co_0_._8_Fe_2_O_4_	35.471	2.5319	0.338	24.41	1.679	8.387	589.956
Cu_0_._3_Co_0_._7_Fe_2_O_4_	35.310	2.5431	0.491	16.79	3.548	8.424	597.799
Cu_0_._4_Co_0_._6_Fe_2_O_4_	35.279	2.5452	0.504	16.36	3.736	8.408	594.399
Cu_0_._5_Co_0_._5_Fe_2_O_4_	35.378	2.5381	0.328	25.14	1.582	8.408	594.399

The XRD analysis was undertaken to examine how progressive Cu substitution modifies the average crystal structure of the spinel ferrite and to establish the structural basis for interpreting the composition-dependent photocatalytic behaviour discussed in subsequent sections.

All diffraction peaks were indexed to the characteristic reflections of cubic spinel ferrite corresponding to the (220), (311), (222), (400), (422), (511), and (440) planes, in good agreement with the standard reference pattern (PDF no. 00-002-1045).^25^ No diffraction peaks associated with crystalline CuO, CoO, Co_3_O_4_, or Fe_2_O_3_ were detected within the instrumental resolution, confirming that all samples retained a single-phase spinel structure throughout the investigated composition range. The absence of detectable secondary phases indicates that Cu substitution modifies the existing spinel lattice without disrupting its overall crystallographic framework, allowing direct comparison of structural evolution among the investigated compositions.

Although the diffraction patterns remained characteristic of spinel ferrite, the principal (311) reflection exhibited a gradual composition-dependent shift (Fig. 2B). The diffraction maximum moved from 35.476° for pristine CFO to values between 35.279° and 35.471° after Cu substitution, with the largest shift toward lower diffraction angle observed for Cu_0_._4_Co_0_._6_Fe_2_O_4_. Accordingly, the corresponding *d*_311_ spacing increased from 2.5316 to 2.5452 Å before decreasing slightly at higher Cu contents (Table 2). The concurrent shift in 2*θ* and expansion of the corresponding *d*_311_ spacing indicate a measurable modification of the average spinel lattice while preserving the cubic structure, consistent with previous reports for Cu-substituted ferrites.^25^ Notably, the peak displacement did not increase proportionally with Cu content. Instead, the largest lattice expansion occurred at intermediate compositions, suggesting that the structural response deviates from a simple linear substitution process.

Peak broadening accompanied the shift of the (311) reflection. The full width at half maximum (FWHM) increased from 0.311° for CFO to 0.504° for Cu_0_._4_Co_0_._6_Fe_2_O_4_, whereas the Scherrer-derived crystallite-size estimate decreased from 26.53 to 16.36 nm (Fig. 2C). The smallest estimates were observed for Cu_0_._3_Co_0_._7_Fe_2_O_4_ and Cu_0_._4_Co_0_._6_Fe_2_O_4_, followed by a slight increase at *x* = 0.5. Because the Scherrer equation estimates coherent diffraction-domain size rather than particle size,^26^ these values are interpreted as comparative indicators within the catalyst series. The (311) reflection was selected because it was the most clearly resolved and consistently identifiable peak across the series. Only this reflection was analysed; therefore, the Scherrer-derived values cannot be used to independently separate crystallite-size and lattice-strain contributions. Williamson–Hall analysis was not applied because a single reflection is insufficient for this purpose. Accordingly, the reported values are treated as relative changes in diffraction-domain size rather than definitive crystallite-size measurements or direct measures of microstrain.

The calculated dislocation density (*δ* = 1/*D*^2^) increased from 1.421 × 10^−3^ to 3.736 × 10^−3^ nm^−2^ between CFO and Cu_0_._4_Co_0_._6_Fe_2_O_4_ (Table 2). Although this parameter does not represent the absolute defect concentration, it is widely employed for comparative assessment of crystallographic disorder in ferrites.^26^ The simultaneous increase in FWHM and dislocation density, together with crystallite refinement, indicates that the average crystal structure experiences its greatest degree of structural perturbation within the intermediate Cu-substituted compositions (*x* = 0.3–0.4). Together, the XRD-derived parameters describe the evolution of the average crystal structure but do not, by themselves, identify the structural characteristics governing the subsequent photocatalytic behaviour.

Further insight into the structural evolution is provided by the lattice parameter and unit-cell volume (Fig. 2D). The lattice parameter increased from 8.386 Å for pristine CFO to a maximum of 8.424 Å for Cu_0_._3_Co_0_._7_Fe_2_O_4_, accompanied by an increase in unit-cell volume from 589.745 to 597.799 Å^3^. At higher Cu contents, both parameters decreased slightly and approached nearly constant values. This non-monotonic trend does not exhibit the linear lattice evolution expected from an ideal Vegard-type relationship, indicating that the average lattice response cannot be described solely by nominal Cu substitution. Instead, the lattice expanded most strongly at intermediate compositions before partially contracting, suggesting that structural adjustment within the spinel lattice follows a non-linear compositional response.

From a crystal-chemical perspective, this behaviour may arise from the combined effects of ionic-size mismatch, local lattice distortion, and changes in cation distribution within the spinel structure.^27^ Cu^2+^ is frequently associated with Jahn–Teller distortion when occupying octahedral sites,^27^ although conventional powder XRD probes only the average long-range crystal structure. Consequently, XRD alone cannot distinguish local distortion, inversion degree, or cation-site occupancy. The present results therefore establish systematic structural evolution across the composition series without identifying its microscopic origin. Clarifying the underlying crystal-chemical changes would require complementary local-structure techniques, such as EXAFS, pair distribution function (PDF) analysis, or neutron diffraction.

The XRD-derived parameters consistently indicate that the intermediate compositions (*x* = 0.3–0.4) exhibit the smallest crystallite sizes, the broadest diffraction peaks, the highest dislocation densities, and the largest lattice dimensions. Together, these observations indicate that structural variation is most pronounced within the intermediate composition range. However, the available XRD data do not permit identification of the specific structural characteristics governing the observed photocatalytic behaviour, because XRD primarily probes the average long-range crystal structure rather than the local atomic environment.

Overall, the XRD results demonstrate that Cu substitution produces a non-linear evolution of the spinel crystal structure while preserving phase purity throughout the investigated composition range. The XRD results therefore provide the crystallographic framework for integrating the spectroscopic, optical, and photocatalytic evidence presented in the following sections.

#### 3.1.2. Composition-dependent evolution of metal–oxygen bonding revealed by FTIR spectroscopy

Following the crystallographic analysis, FTIR spectroscopy was employed to examine how Cu substitution affects the metal–oxygen vibrational characteristics of the spinel ferrite framework. The spectra of pristine CoFe_2_O_4_ and Cu_*x*_Co_1−*x*_Fe_2_O_4_ (*x* = 0–0.5) are presented in Fig. 3A.

**Fig. 3:**
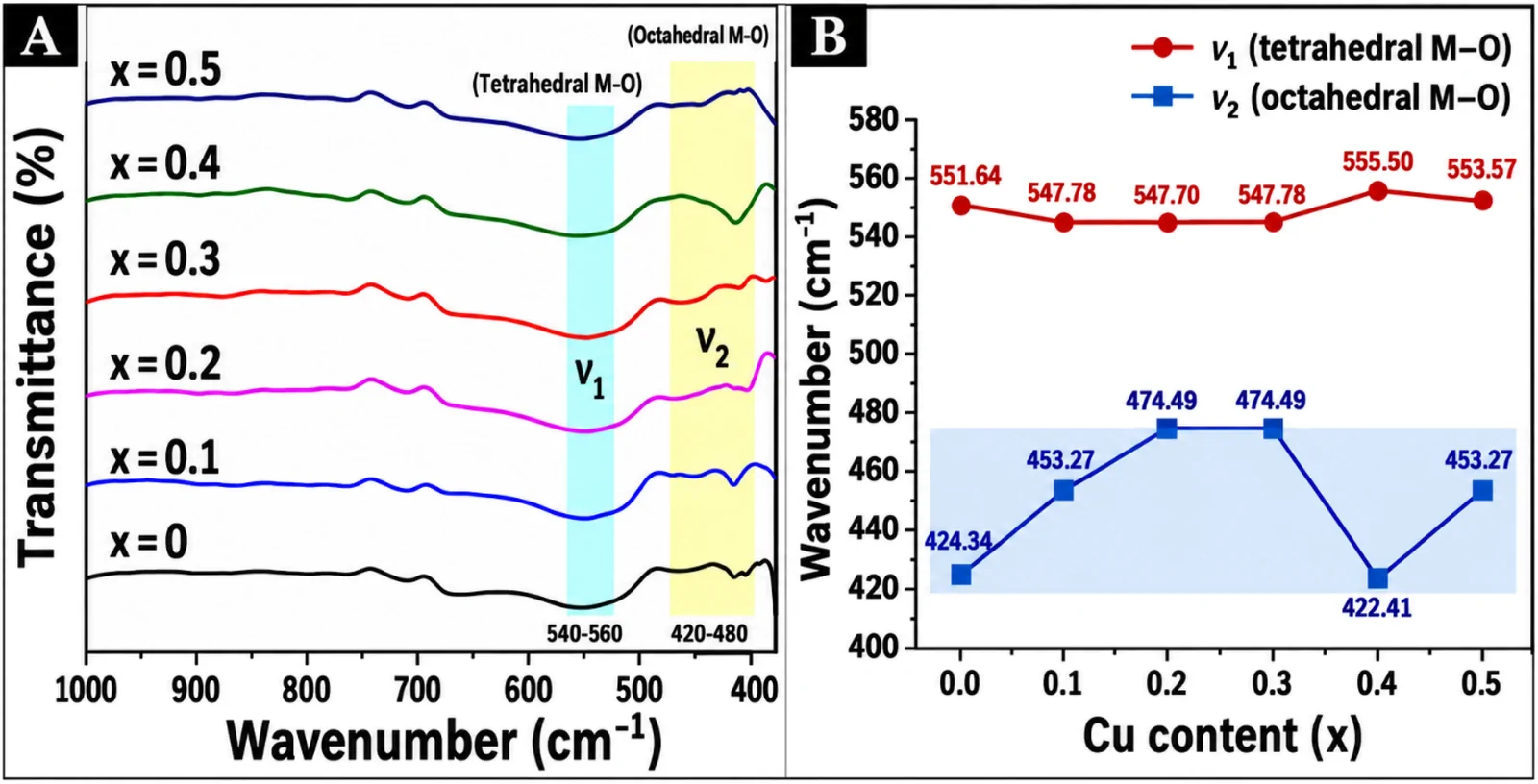
FTIR analysis of Cu_*x*_Co_1−*x*_Fe_2_O_4_ ferrites (*x* = 0–0.5): (A) FTIR spectra showing the characteristic tetrahedral (*ν*_1_) and octahedral (*ν*_2_) metal–oxygen vibration bands; (B) evolution of *ν*_1_ and *ν*_2_ band positions as a function of Cu content.

All compositions exhibit two characteristic absorption bands centred at 547–556 cm^−1^ (*ν*_1_) and 422–474 cm^−1^ (*ν*_2_), corresponding predominantly to metal–oxygen stretching vibrations associated with the tetrahedral and octahedral sublattices, respectively. The persistence of both bands throughout the substitution series is consistent with retention of the fundamental spinel framework, in agreement with the phase-purity results obtained by XRD. Although the overall spectral profiles remain similar, the two vibration bands evolve differently with Cu content (Fig. 3B). The *ν*_1_ band decreases modestly from 551.64 cm^−1^ for pristine CoFe_2_O_4_ to approximately 547.7 cm^−1^ for *x* = 0.1–0.3 before shifting toward higher wavenumbers at *x* = 0.4–0.5. In contrast, *ν*_2_ exhibits a substantially larger variation, increasing from 424.34 to 474.49 cm^−1^ before returning to lower values at higher Cu contents. These composition-dependent shifts are consistent with changes in the metal–oxygen vibrational characteristics of the ferrite framework, but they do not by themselves establish the occupancy of specific cations within the tetrahedral or octahedral sublattices.

The larger variation observed for *ν*_2_ is therefore discussed as a change in the vibrational response associated with the octahedral sublattice rather than as direct evidence of a specific cation redistribution. Peak broadening and band overlap in mixed-metal spinel ferrites further limit the assignment of individual metal–oxygen contributions. Accordingly, the measured *ν*_2_ position should be regarded as a descriptor of the overall metal–oxygen vibrational characteristics rather than a direct measure of an individual metal–oxygen bond or a specific cation-site occupancy.

Ferrite vibration frequencies can be influenced by several factors, including bond length, bond strength, local coordination geometry, and cation occupancy. Because these factors may evolve concurrently during Cu substitution, individual band shifts cannot be uniquely assigned to a single structural variable. Thus, any association between the observed FTIR shifts and changes in Cu/Co/Fe site preference is treated here as a hypothesis rather than as established structural evidence. Definitive determination of site-specific cation occupancy would require more directly site-sensitive characterization, such as extended X-ray absorption fine structure (EXAFS) spectroscopy or pair distribution function (PDF) analysis.

Comparison of the FTIR and XRD datasets shows that both techniques capture composition-dependent structural changes, although they probe different structural length scales. The largest *ν*_2_ displacement, reaching approximately 50 cm^−1^ relative to pristine CoFe_2_O_4_, occurs within the intermediate composition interval (*x* = 0.2–0.3), where XRD also records pronounced changes in lattice parameter, crystallite size, and apparent dislocation density. In contrast, *ν*_1_ reaches its minimum position at lower Cu contents (*x* ≈ 0.1–0.3), suggesting that the vibrational responses associated with the tetrahedral and octahedral sublattices do not evolve synchronously during progressive Cu substitution. These differences suggest that local structural adjustment proceeds through multiple coordination environments rather than through a uniform modification of the spinel lattice.

Viewed together, the XRD and FTIR results describe the structural evolution of Cu_*x*_Co_1−*x*_Fe_2_O_4_ at two complementary length scales. XRD captures changes in the average crystallographic framework, whereas FTIR reflects variations in the local metal–oxygen bonding environment. Both techniques consistently indicate that the most pronounced structural changes occur within the intermediate composition range while confirming preservation of the spinel framework throughout the substitution series. This convergence strengthens confidence in the observed structural evolution without implying that either technique alone identifies the structural characteristics governing photo-Fenton performance.

Within this context, FTIR completes the structural characterization by linking the average crystallographic evolution identified by XRD with composition-dependent changes in local metal–oxygen bonding. The following section examines whether these structural variations are accompanied by corresponding changes in the optical properties of the ferrite series.

#### 3.1.3. Morphological and elemental characterization by SEM, TEM, and EDS

SEM and TEM images of pristine CoFe_2_O_4_ and Cu_0_._4_Co_0_._6_Fe_2_O_4_ are presented in Fig. 4.

**Fig. 4:**
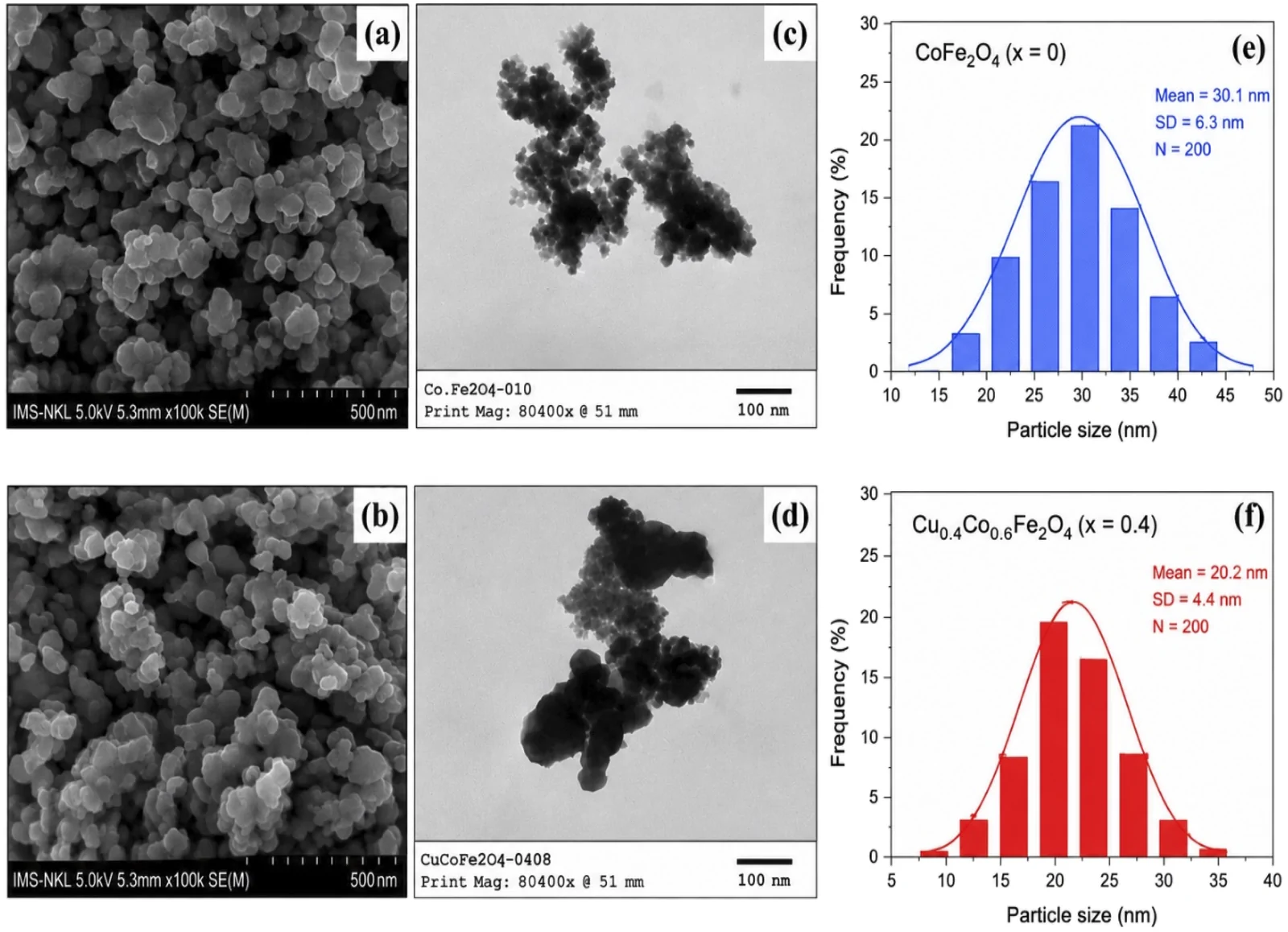
Morphological characterization of CoFe_2_O_4_ and Cu_0_._4_Co_0_._6_Fe_2_O_4_: (a and b) SEM images; (c and d) TEM images; (e and f) particle-size distribution histograms determined from TEM images by measuring the equivalent circular diameter (ECD) of individual primary nanoparticles (*N* = 200 for each sample).

Both samples consist of nanosized particles with predominantly quasi-spherical to irregular polyhedral morphologies, together with a moderate degree of interparticle aggregation. Within the resolution limits of the SEM observations, Cu substitution does not induce obvious changes in particle morphology or apparent aggregation behavior. This observation agrees with the XRD and FTIR analyses, which indicate that Cu incorporation modifies the crystal structure while preserving the spinel framework. The structural evolution therefore appears to occur without large-scale morphological reconstruction of the ferrite particles.

A more pronounced effect of Cu substitution is observed in the particle dimensions. To obtain a statistically representative evaluation, particle-size distributions were determined directly from TEM micrographs by measuring the equivalent circular diameter (ECD) of individual primary nanoparticles. Approximately 200 particles were selected from multiple randomly chosen, non-overlapping TEM regions for each sample to minimize sampling bias. The resulting particle-size distributions show approximately symmetrical profiles centered around the mean particle diameters, yielding average particle sizes of 30.1 ± 6.3 nm for pristine CoFe_2_O_4_ and 20.2 ± 4.4 nm for Cu_0_._4_Co_0_._6_Fe_2_O_4_. The TEM results therefore indicate that Cu substitution decreases the average nanoparticle diameter by approximately 33%. XRD reveals a comparable reduction in crystallite size from 26.53 to 16.36 nm (≈38%). Although TEM and XRD probe different structural characteristics, with TEM measuring physical particle dimensions and XRD estimating coherent diffraction-domain sizes, their consistent trends indicate that Cu incorporation favors the formation of smaller ferrite nanoparticles under the present synthesis conditions.

Partial aggregation is evident in both the SEM and TEM images (Fig. 4). However, the aggregates are composed of distinguishable primary nanoparticles rather than fused grains, indicating that interparticle association dominates over extensive grain coalescence. Therefore, the histograms in Fig. 4e and f represent the size distribution of individual primary nanoparticles rather than the dimensions of the secondary aggregates visible in the SEM images.

The EDS spectra (Fig. 5) are consistent with the intended compositions of the synthesized ferrites. Pristine CoFe_2_O_4_ exhibits only Co, Fe, and O signals, whereas Cu_0_._4_Co_0_._6_Fe_2_O_4_ additionally displays characteristic Cu signals without detectable impurity elements within the sensitivity of the technique. These observations suggest that Cu incorporation does not introduce detectable secondary elemental contaminants within the analytical limitations of EDS.

**Fig. 5:**
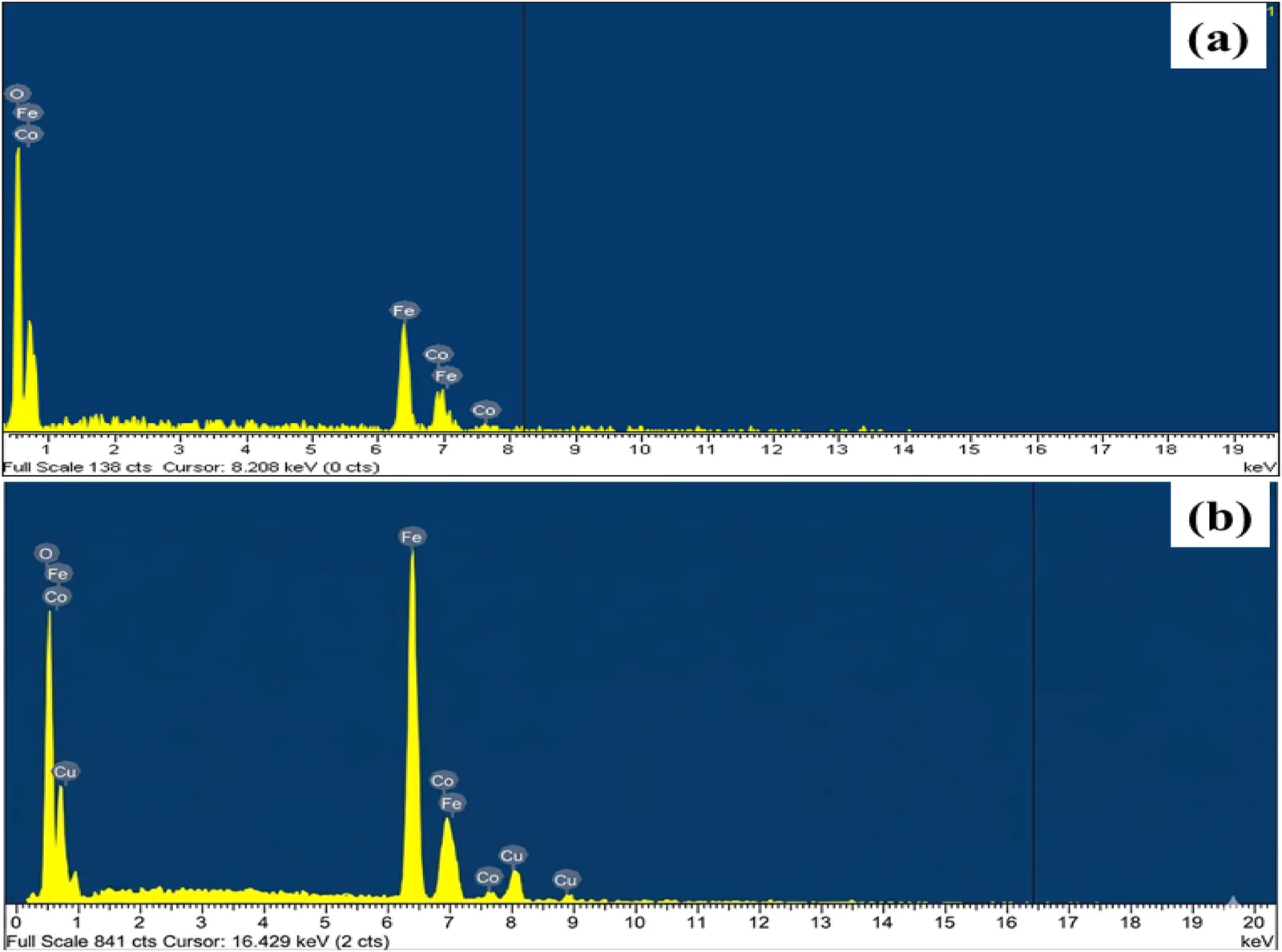
Energy-dispersive X-ray spectroscopy (EDS) spectra of (a) CoFe_2_O_4_ and (b) Cu_0_._4_Co_0_._6_Fe_2_O_4_ nanoparticles.

The semi-quantitative EDS analysis (Table S1, (SI)) follows the nominal compositional trend, supporting successful Cu incorporation while preserving the principal ferrite constituents. Minor deviations in the elemental ratios are expected from the semi-quantitative nature of EDS analysis, particularly for light elements such as oxygen, and may additionally reflect local compositional heterogeneity, particle aggregation, and surface-related effects. Within these analytical limitations, the EDS results remain consistent with the intended chemical compositions of the synthesized ferrites.

#### 3.1.4. Surface chemical states revealed by X-ray photoelectron spectroscopy

X-ray photoelectron spectroscopy (XPS) was used to characterize the surface chemical states of pristine Cu_0_._4_Co_0_._6_Fe_2_O_4_ (Fig. 6).

**Fig. 6:**
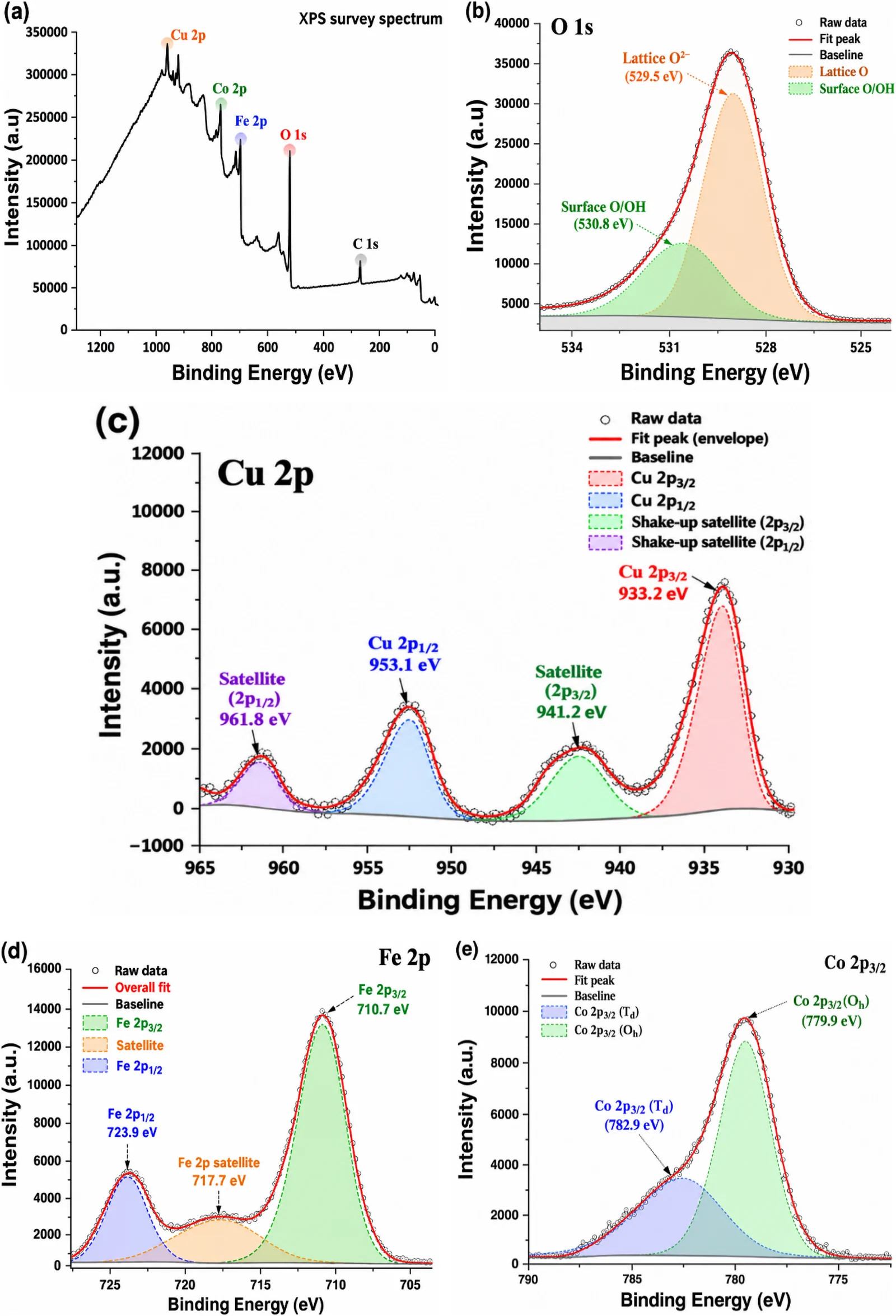
XPS characterization of pristine Cu_0_._4_Co_0_._6_Fe_2_O_4_. (a) Survey spectrum and high-resolution spectra of (b) O 1s, (c) Cu 2p, (d) Fe 2p, and (e) Co 2p, with corresponding fitted components.

The survey spectrum (Fig. 6a) shows characteristic Cu, Co, Fe, and O signals, together with a C 1s contribution attributable to adventitious carbon, consistent with the expected surface elemental composition. High-resolution spectra further resolve the chemical states of O and the transition-metal cations (Fig. 6b–e).

The O 1s profile (Fig. 6b) is dominated by lattice O^2−^ at 529.5 eV, with a higher-binding-energy contribution at 530.8 eV associated with surface oxygen-containing species. This distribution is consistent with the metal–oxygen-related vibrations observed by FTIR. The Cu 2p spectrum (Fig. 6c) exhibits Cu 2p_3/2_ and Cu 2p_1/2_ components at 933.2 and 953.1 eV, respectively, with a spin–orbit separation of 19.9 eV. Pronounced shake-up satellites at 941.2 and 961.8 eV are characteristic of Cu^2+^ and are consistent with reported Cu-containing spinel oxides.^28,29^ Together, these features support Cu^2+^ as the predominant surface Cu oxidation state.

The Fe 2p spectrum (Fig. 6d) shows Fe 2p_3/2_ and Fe 2p_1/2_ components at 710.7 and 723.9 eV, respectively, together with a satellite at 717.7 eV. The spectral profile is consistent with Fe in an oxide environment, while the satellite is consistent with the multiplet/shake-up structure commonly observed for Fe oxides. The Co 2p_3/2_ region (Fig. 6e) was fitted with components at 779.9 and 782.9 eV, reflecting overlapping contributions within the surface Co 2p envelope.

Overall, the XPS results provide complementary surface-sensitive evidence for the chemical states of Cu, Co, Fe, and O in Cu_0_._4_Co_0_._6_Fe_2_O_4_. However, XPS alone does not unambiguously determine the distribution of Cu, Co, and Fe between tetrahedral and octahedral spinel sites. Accordingly, the present XPS results are used to characterize surface chemical states rather than to assign crystallographic cation-site occupancy. Definitive determination of site-specific cation distribution would require more directly site-sensitive techniques, such as neutron diffraction or element-specific X-ray absorption spectroscopy.

#### 3.1.5. Surface area and pore structure analysis

Representative N_2_ adsorption–desorption isotherms together with the corresponding textural parameters are presented in Fig. 7 and Tables 3, S2 (SI). Both samples exhibit limited N_2_ uptake over most of the relative-pressure range, followed by a sharp increase as *P*/*P*_0_ approaches unity. This adsorption behaviour is consistent with capillary condensation occurring predominantly within interparticle voids rather than within an extensively developed microporous network.

**Fig. 7:**
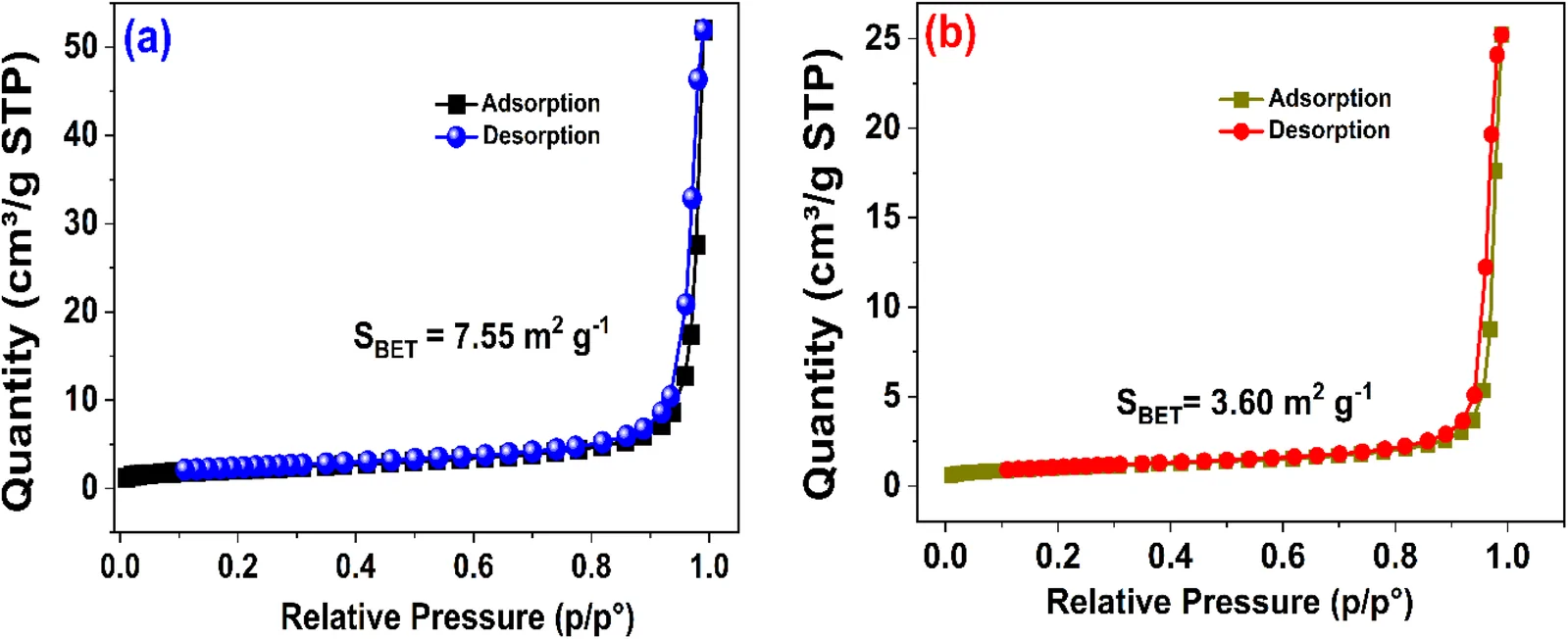
N_2_ adsorption–desorption analysis of ferrite samples. (a) BET linear fitting region of CoFe_2_O_4_. (b) N_2_ adsorption–desorption isotherm of Cu_0_._4_Co_0_._6_Fe_2_O_4_ measured at 77 K.

**Table 3:** Textural properties of CoFe_2_O_4_ and Cu_0_._4_Co_0_._6_Fe_2_O_4_ derived from N_2_ adsorption–desorption measurements

Sample	BET surface area (m^2^ g^−1^)	Total pore volume (cm^3^ g^−1^)	Average pore diameter (nm)
CoFe_2_O_4_	7.55	0.0797	50.07
Cu_0_._4_Co_0_._6_Fe_2_O_4_	3.60	0.0386	56.04

The BJH analysis gives average adsorption pore widths of 50.07 nm for pristine CoFe_2_O_4_ and 56.04 nm for Cu_0_._4_Co_0_._6_Fe_2_O_4_, while the corresponding desorption pore width increases from 41.29 to 43.82 nm. These pore widths lie near the upper boundary of the mesoporous regime and are consistent with the adsorption–desorption profiles, indicating that the accessible pore system is governed predominantly by interparticle voids rather than by intrinsic framework porosity. The similar adsorption–desorption profiles further indicate that Cu substitution modifies the dimensions of the accessible pore network without fundamentally changing its structural character.

Cu substitution is accompanied by a marked reduction in accessible surface area and pore volume. The BET surface area decreases from 7.55 to 3.60 m^2^ g^−1^, corresponding to a reduction of approximately 52.3%, while the BJH adsorption pore volume decreases from 0.0797 to 0.0386 cm^3^ g^−1^ (−51.6%). By contrast, the average adsorption pore width increases from 50.07 to 56.04 nm (+11.9%). This combination of trends suggests that the decrease in accessible surface is associated primarily with a reduction in pore volume rather than progressive pore constriction. Instead, the remaining pore network becomes moderately coarser while occupying a smaller accessible volume.

Further insight is obtained from the *t*-plot analysis. The micropore surface area changes only slightly, decreasing from 0.90 to 0.86 m^2^ g^−1^ (−4.4%), whereas the external surface area declines from 6.65 to 2.74 m^2^ g^−1^ (−58.8%). Accordingly, the reduction in BET surface area is governed primarily by the externally accessible surface rather than by changes in the already limited microporous fraction. This interpretation is supported by the relative contribution of the external surface, which accounts for approximately 88% of the total BET surface area in pristine CoFe_2_O_4_ and 76% after Cu substitution. Collectively, these observations suggest that Cu incorporation predominantly reorganizes the external surface architecture while leaving the microporous contribution largely unaffected.

The combined BET, BJH, and *t*-plot results suggest that Cu substitution modifies surface accessibility without fundamentally altering the architectural characteristics of the pore network. Although the accessible surface area and pore volume decrease by approximately one half, the mesoporous character is retained and the average pore width increases modestly. These observations are not consistent with complete pore collapse or loss of pore connectivity. Rather, they are consistent with a redistribution of accessible interparticle void space while preserving the overall pore architecture probed by N_2_ adsorption.

The origin of these textural changes cannot be resolved from adsorption measurements alone. Variations in particle packing, aggregate arrangement, and interparticle spacing may each contribute to the observed reduction in accessible surface area. The microscopy observations support this interpretation by revealing aggregated assemblies composed of discrete primary nanoparticles rather than consolidated grains. Within the limits of the present dataset, therefore, the adsorption results are most appropriately interpreted as reflecting changes in external surface accessibility rather than direct modification of the intrinsic pore structure of individual ferrite particles.

Considered together with the XRD, FTIR, and microscopy analyses, the adsorption data complete the characterization of the Cu_*x*_Co_1−*x*_Fe_2_O_4_ series. Diffraction, vibrational spectroscopy, electron microscopy, and gas adsorption each probe different material characteristics, spanning the average crystallographic framework, the local metal–oxygen environment, particle architecture, and surface accessibility, respectively. Viewed collectively, these complementary observations establish that Cu substitution influences several material characteristics simultaneously rather than modifying a single structural descriptor. This provides the framework for examining the optical evolution of the ferrite series in the following section.

#### 3.1.6. Optical absorption properties and band-gap analysis

Diffuse reflectance spectra expressed as the Kubelka–Munk function together with the corresponding Tauc-derived band-gap energies are presented in Fig. 8, 9 and Table 4. All compositions exhibit broad optical absorption extending from the ultraviolet into the visible region, with relatively diffuse absorption edges characteristic of gradual optical transitions in spinel ferrites containing multiple transition-metal cations.

**Fig. 8:**
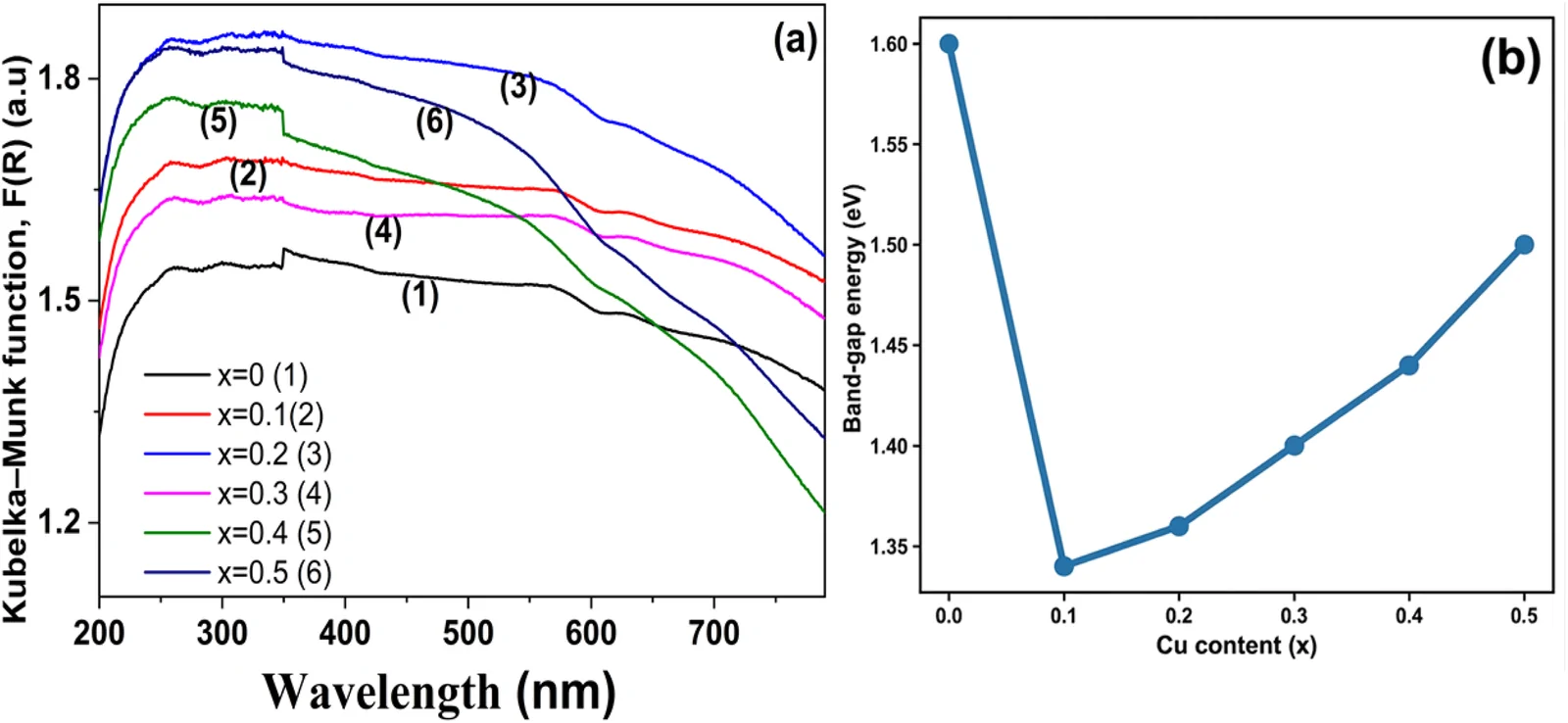
(a) Diffuse reflectance spectra expressed as the Kubelka–Munk function, *F*(*R*), for the Cu_*x*_Co_1−*x*_Fe_2_O_4_ series (*x* = 0–0.5). (b) Composition-dependent variation of the optical band-gap energy (*E*_g_) obtained from Tauc analysis of the Kubelka–Munk-transformed diffuse reflectance spectra.

**Fig. 9:**
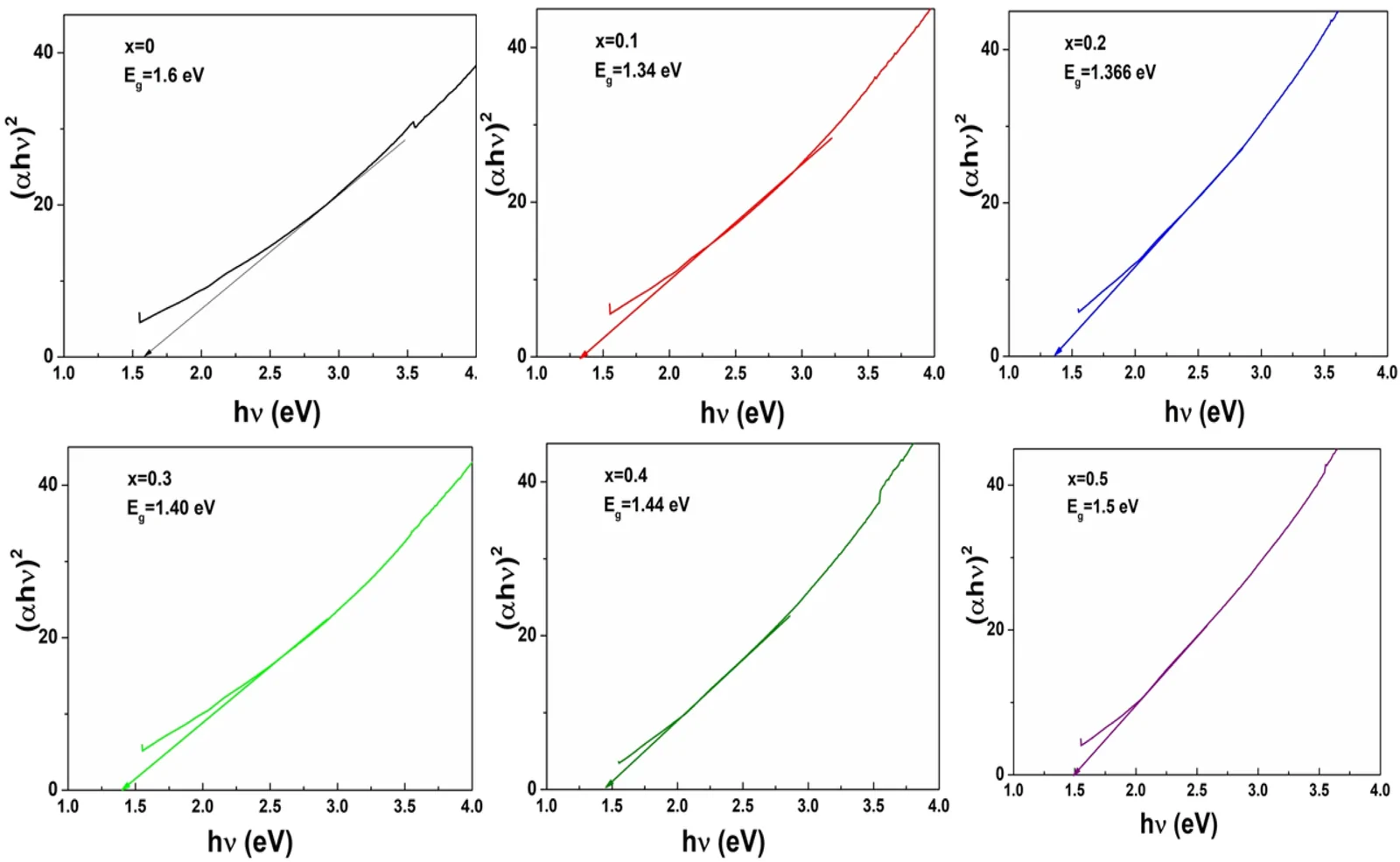
Tauc plots derived from Kubelka–Munk-transformed diffuse reflectance spectra for Cu_*x*_Co_1−*x*_Fe_2_O_4_ (*x* = 0–0.5). The optical band gaps were estimated by extrapolating the linear region of the (*F*(*R*)*hν*)^2^*versus hν* plots to the photon-energy axis.

**Table 4:** Optical band-gap energies of Cu_*x*_Co_1−*x*_Fe_2_O_4_ estimated from Tauc plots derived from Kubelka–Munk-transformed diffuse reflectance spectra

Sample	Cu content, *x*	*E*_g_ (eV)	Δ*E*_g_ relative to CoFe_2_O_4_ (%)
CoFe_2_O_4_	0.0	1.60	0.0
Cu_0_._1_Co_0_._9_Fe_2_O_4_	0.1	1.34	−16.3
Cu_0_._2_Co_0_._8_Fe_2_O_4_	0.2	1.36	−15.0
Cu_0_._3_Co_0_._7_Fe_2_O_4_	0.3	1.40	−12.5
Cu_0_._4_Co_0_._6_Fe_2_O_4_	0.4	1.44	−10.0
Cu_0_._5_Co_0_._5_Fe_2_O_4_	0.5	1.50	−6.3

Although the overall spectral profiles remain similar across the composition series, the band-gap energy varies systematically with Cu content. Pristine CoFe_2_O_4_ exhibits an optical band gap (*E*_g_) of 1.60 eV, whereas Cu incorporation lowers *E*_g_ to 1.34 eV at *x* = 0.1, corresponding to a reduction of approximately 16.3%. Further increases in Cu content are accompanied by a gradual recovery of the band gap to 1.36, 1.40, 1.44, and 1.50 eV for *x* = 0.2–0.5, respectively. Despite this recovery, every Cu-substituted composition retains a lower *E*_g_ than pristine CoFe_2_O_4_ throughout the investigated compositional range.

The variation in *E*_g_ does not follow a linear dependence on Cu content. The largest reduction occurs at the lowest substitution level (*x* = 0.1), after which the band gap increases progressively with further Cu incorporation while remaining below that of pristine CoFe_2_O_4_. The total variation spans 0.26 eV across the investigated composition range, from 1.60 eV for CoFe_2_O_4_ to 1.34 eV for Cu_0_._1_Co_0_._9_Fe_2_O_4_. This non-monotonic response indicates that optical evolution does not scale directly with Cu concentration. Instead, relatively small compositional changes are accompanied by measurable differences in the electronic transitions probed by diffuse reflectance spectroscopy. The present optical data do not identify the origin of the band-gap variation, nor do they distinguish among the possible electronic or structural contributions responsible for the observed trend. Accordingly, the DRS results are most appropriately interpreted as experimental evidence of composition-dependent optical evolution rather than direct evidence of any specific electronic mechanism.

Comparison with the preceding characterization results reveals that the optical response follows a compositional trend distinct from those observed for the crystallographic, morphological, and textural characteristics. The minimum band-gap energy occurs at *x* = 0.1, whereas the largest crystallographic variation, particle refinement, and surface reorganization are observed within the intermediate Cu compositions. This divergence indicates that the optical response cannot be interpreted solely from any individual structural characteristic measured in the present work. Instead, the available data indicate that optical evolution accompanies multiple composition-dependent structural changes, although the present measurements do not permit the relative contribution of each factor to be distinguished.

The DRS analysis demonstrates that Cu substitution also modifies the optical response of the ferrite series, with measurable yet non-linear variation across the investigated composition range. Importantly, the composition exhibiting the smallest optical band gap (*x* = 0.1) does not coincide with that exhibiting the highest photo-Fenton activity. Cu_0_._1_Co_0_._9_Fe_2_O_4_ exhibits the minimum *E*_g_ of 1.34 eV, whereas Cu_0_._4_Co_0_._6_Fe_2_O_4_, with a slightly higher *E*_g_ of 1.44 eV, exhibits the highest apparent rate constant. This divergence suggests that band-gap narrowing alone is insufficient to predict catalytic performance. Although a lower *E*_g_ reduces the energetic threshold for photoexcitation, the overall photo-Fenton rate also depends on subsequent interfacial redox processes. Notably, Cu_0_._4_Co_0_._6_Fe_2_O_4_ exhibits a smaller crystallite size and greater diffraction-line broadening than Cu_0_._1_Co_0_._9_Fe_2_O_4_, while retaining a band gap compatible with visible-light excitation.

Thus, the superior activity of Cu_0_._4_Co_0_._6_Fe_2_O_4_ cannot be attributed to band-gap energy alone. Rather, the results support a composition-dependent interplay between optical and structural characteristics. Because charge-carrier dynamics were not directly measured in this study, the present data do not permit identification of the specific electronic process underlying the activity maximum. The relationship between these composition-dependent optical characteristics and photo-Fenton performance is examined further in the following section.

### 3.2. Photo-Fenton degradation performance, process optimization, and mineralization assessment

#### 3.2.1. Dark adsorption and equilibration prior to photo-Fenton experiments

The dark adsorption profiles of Rhodamine B (RhB) on the Cu_*x*_Co_1−*x*_Fe_2_O_4_ catalysts (*x* = 0–0.5) are presented in Fig. 10.

**Fig. 10:**
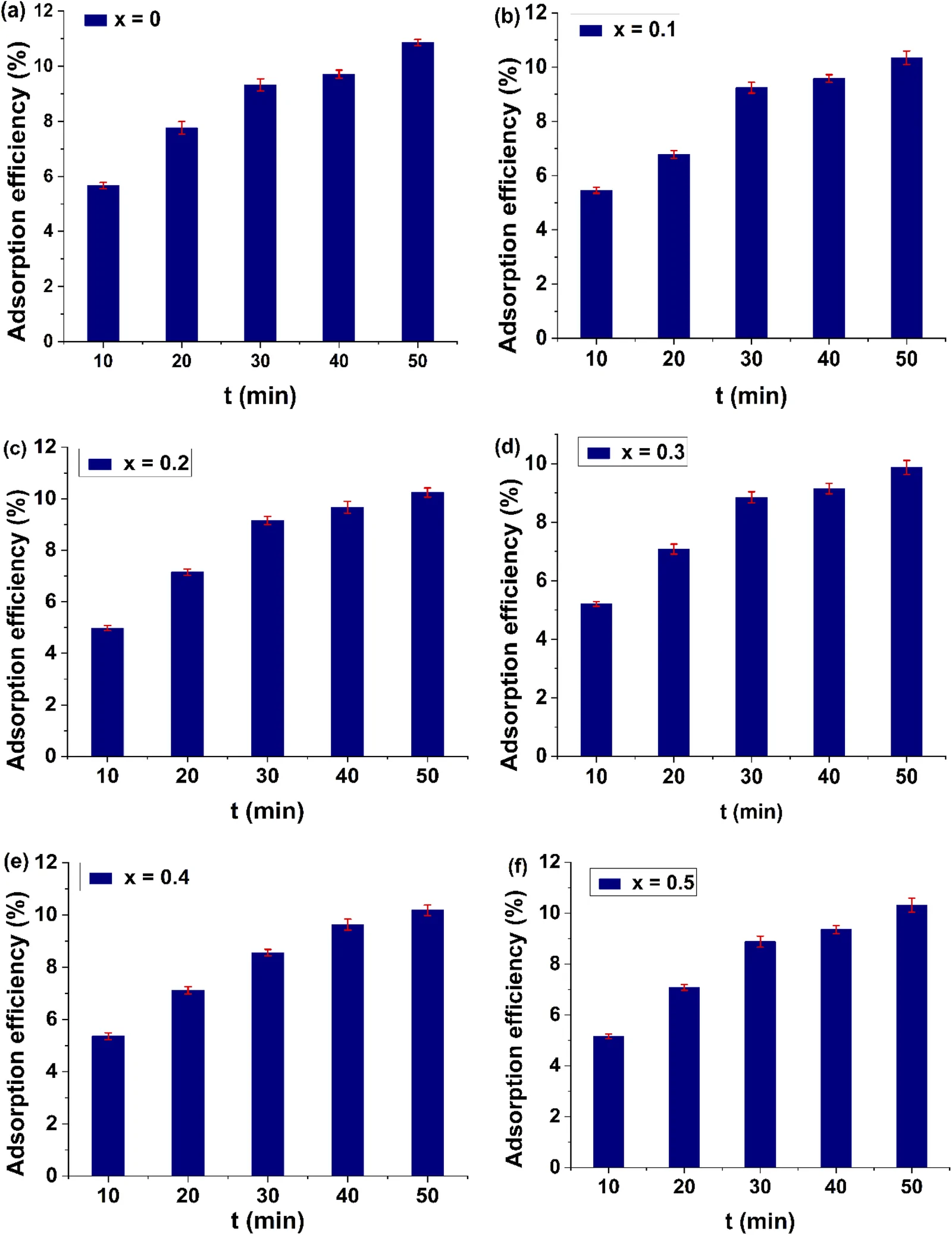
Dark adsorption of Rhodamine B (RhB) on Cu*_x_*Co_1−*x*_Fe_2_O_4_ catalysts as a function of contact time: (a) *x* = 0, (b) *x* = 0.1, (c) *x* = 0.2, (d) *x* = 0.3, (e) *x* = 0.4, and (f) *x* = 0.5. Values are means of triplicate measurements (*n* = 3); error bars represent standard deviations.

For all compositions, RhB adsorption increased with contact time, particularly during the first 30 min. At 30 min, the adsorption efficiencies ranged from 8.56% to 9.32%. Extending the contact time to 50 min resulted in only a modest further increase, with adsorption efficiencies reaching 9.87–10.86%. Thus, extending the contact time from 30 to 50 min increased adsorption by only 1.0–1.6 percentage points, indicating that the adsorption rate decreased substantially after 30 min. For the optimized Cu_0_._4_Co_0_._6_Fe_2_O_4_ catalyst, the dark adsorption efficiency remained within this low range, comparable to those of the other compositions. The consistently low adsorption efficiencies (<11%) and the small differences among the Cu_*x*_Co_1−*x*_Fe_2_O_4_ compositions indicate that dark adsorption makes only a limited contribution to the overall RhB removal during the subsequent photo-Fenton process. The similar adsorption behaviour across the composition series provides a sufficiently comparable initial adsorption baseline before visible-light irradiation. A 30 min dark equilibration period was therefore selected as a practical and consistent pre-irradiation condition for all subsequent photo-Fenton experiments.

This procedure limits further adsorption during irradiation, allowing subsequent differences in RhB removal to be compared primarily in terms of photo-Fenton performance under comparable pre-irradiation conditions.

#### 3.2.2. Effect of Cu composition on visible-light photo-Fenton degradation of RhB

The visible-light photo-Fenton performance of the Cu_*x*_Co_1−*x*_Fe_2_O_4_ series is summarized in Fig. 11–13. During irradiation, the characteristic RhB absorption band centred at approximately 554 nm decreases progressively for all compositions, although the rate and extent of absorbance loss vary markedly with Cu content, indicating marked differences in catalytic behaviour among the investigated compositions.

**Fig. 11:**
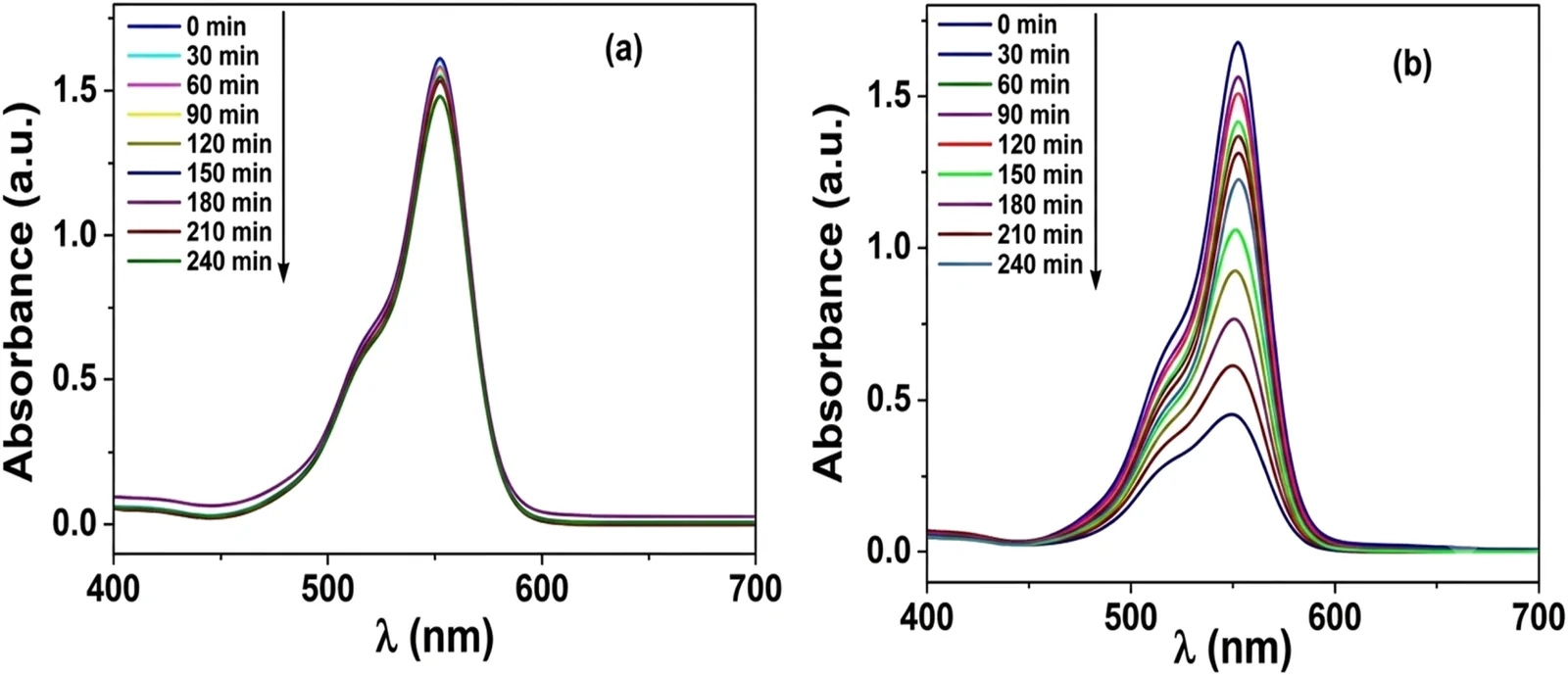
UV-vis spectral evolution of rhodamine B during visible-light irradiation in the presence of (a) H_2_O_2_ alone and (b) CoFe_2_O_4_ alone. The arrow indicates increasing irradiation time (0–240 min).

**Fig. 12:**
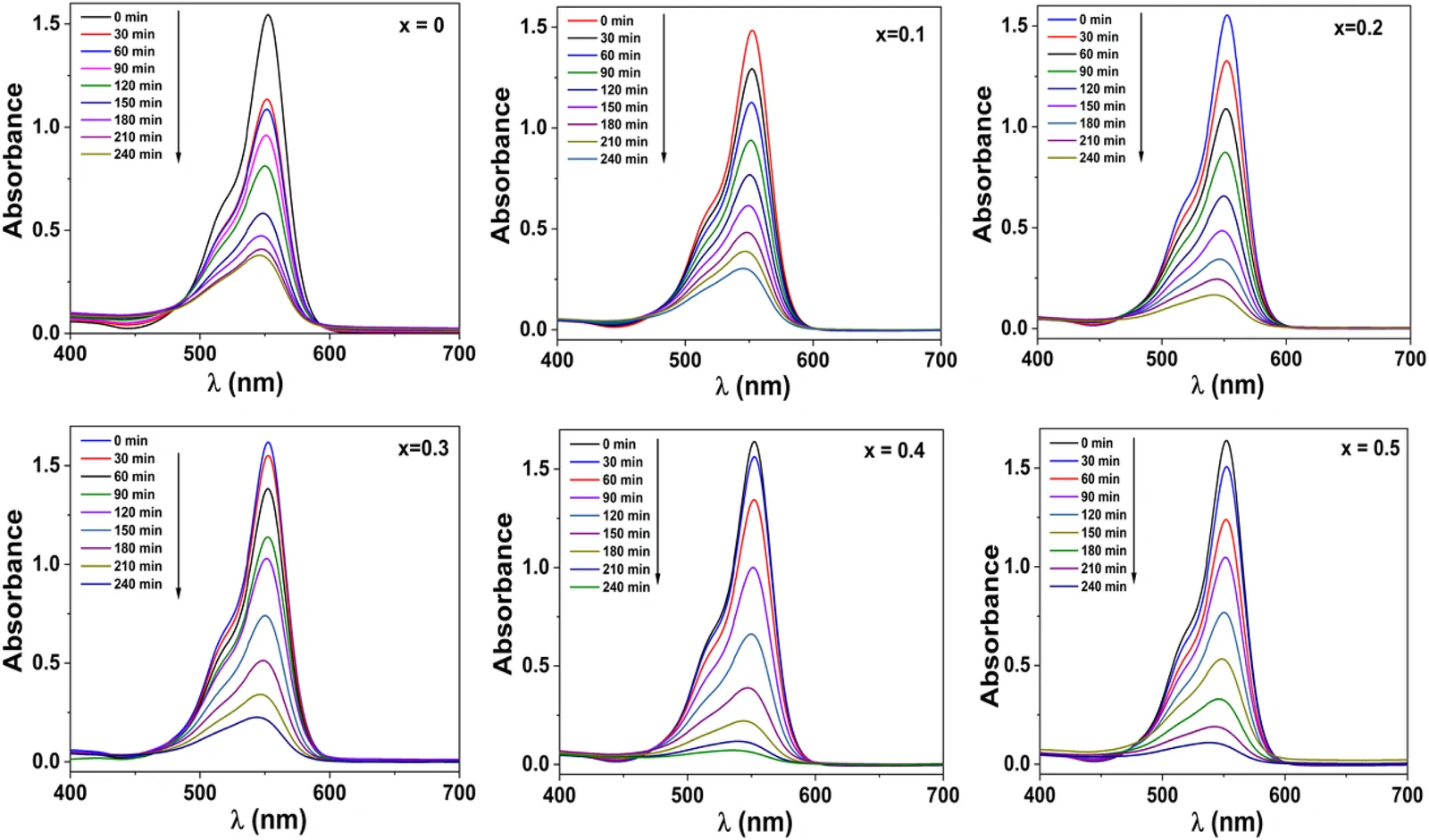
UV-vis spectral evolution of rhodamine B during visible-light photo-Fenton degradation over Cu_*x*_Co_1−*x*_Fe_2_O_4_ catalysts (*x* = 0–0.5) in the presence of H_2_O_2_. The arrow indicates increasing irradiation time (0–240 min).

**Fig. 13:**
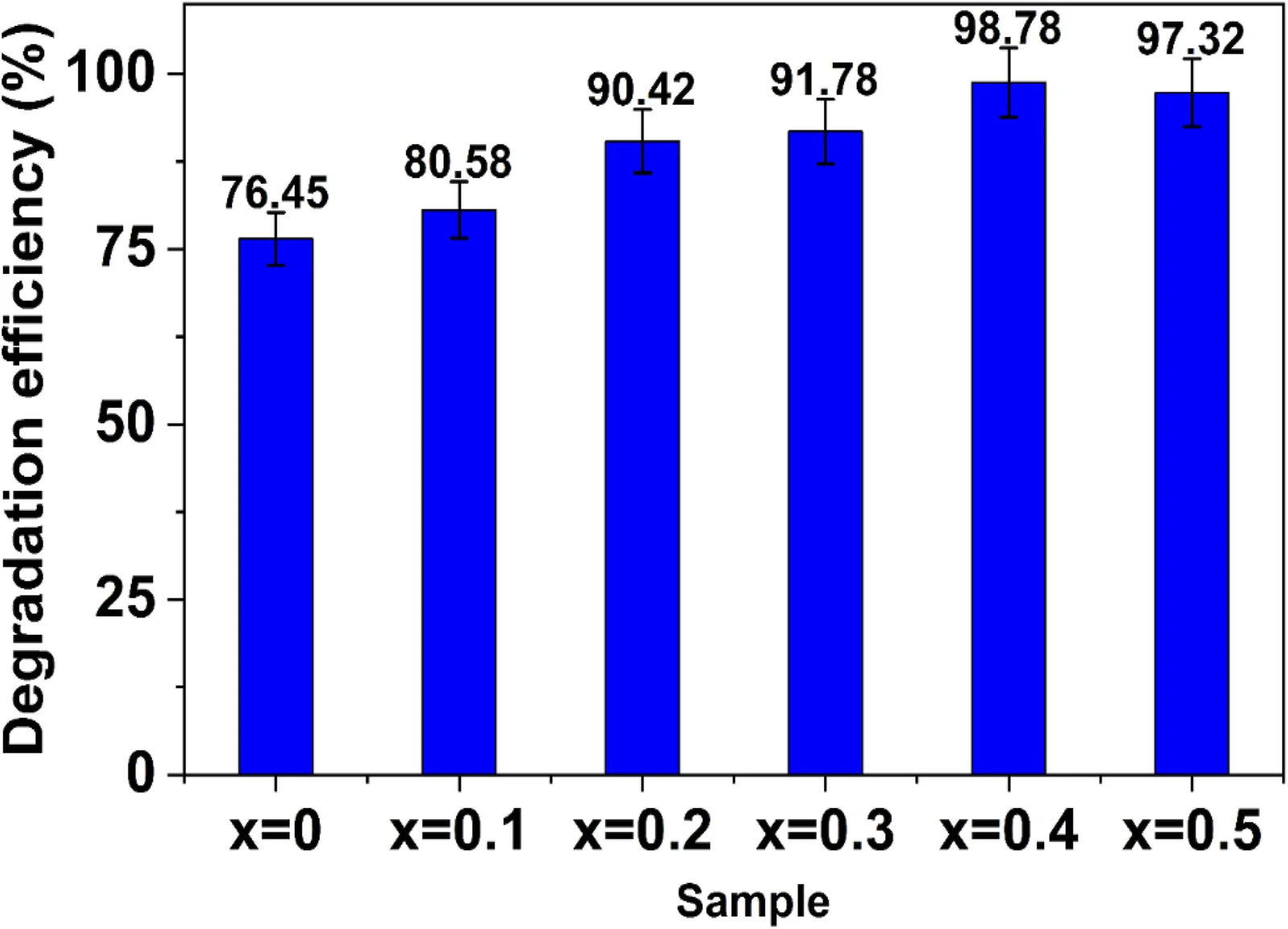
Effect of Cu content on the degradation efficiency of rhodamine B over Cu_*x*_Co_1−*x*_Fe_2_O_4_ catalysts (*x* = 0–0.5) in the presence of H_2_O_2_ after 240 min of visible-light irradiation.

Reference experiments provide a basis for evaluating the respective roles of the catalyst and oxidant. In the presence of H_2_O_2_ alone, RhB removal reaches only 12.17% after 240 min. Visible-light irradiation over pristine CoFe_2_O_4_ without H_2_O_2_ increases removal to 27.28%, whereas combining CoFe_2_O_4_ with H_2_O_2_ increases the degradation efficiency to 76.45%. This value is substantially higher than that obtained with either H_2_O_2_ or CoFe_2_O_4_ alone, indicating that both the catalyst and H_2_O_2_ are required to achieve efficient RhB degradation under the adopted experimental conditions.

Following Cu incorporation, catalytic performance exhibits a clear but non-monotonic compositional dependence. RhB degradation increases from 76.45% for pristine CoFe_2_O_4_ to 80.58%, 90.42%, 91.78%, and 98.78% for *x* = 0.1, 0.2, 0.3, and 0.4, respectively, before decreasing slightly to 97.32% at *x* = 0.5. Relative to pristine CoFe_2_O_4_, the optimum composition achieves a 29.2% increase in degradation efficiency. The existence of a distinct maximum at *x* = 0.4, despite continued Cu incorporation, indicates that catalytic performance is optimized within a relatively narrow compositional interval rather than improving continuously with increasing Cu content.

Comparison with the physicochemical characterization reveals that the catalytic response follows a compositional trend different from those observed for the individual structural and optical characteristics. The minimum optical band gap occurs at *x* = 0.1, whereas the highest degradation efficiency is achieved at *x* = 0.4. This divergence indicates that photocatalytic performance cannot be represented by any single physicochemical parameter measured in the present study. The relationship among composition, physicochemical characteristics, and catalytic behaviour is examined in the integrated discussion presented in Section 3.5.1.

Among the investigated compositions, Cu_0_._4_Co_0_._6_Fe_2_O_4_ consistently exhibits the highest RhB degradation efficiency and was therefore selected for subsequent investigations, including process optimization, mineralization analysis, kinetic evaluation, and reactive-species trapping experiments.

#### 3.2.3. Effect of H_2_O_2_ dosage

Because Cu_0_._4_Co_0_._6_Fe_2_O_4_ exhibited the highest visible-light photo-Fenton activity among the investigated compositions (Section 3.2.2), this catalyst was selected to evaluate the influence of H_2_O_2_ dosage. The corresponding UV-vis spectral evolution and RhB degradation efficiencies are presented in Fig. 14.

**Fig. 14:**
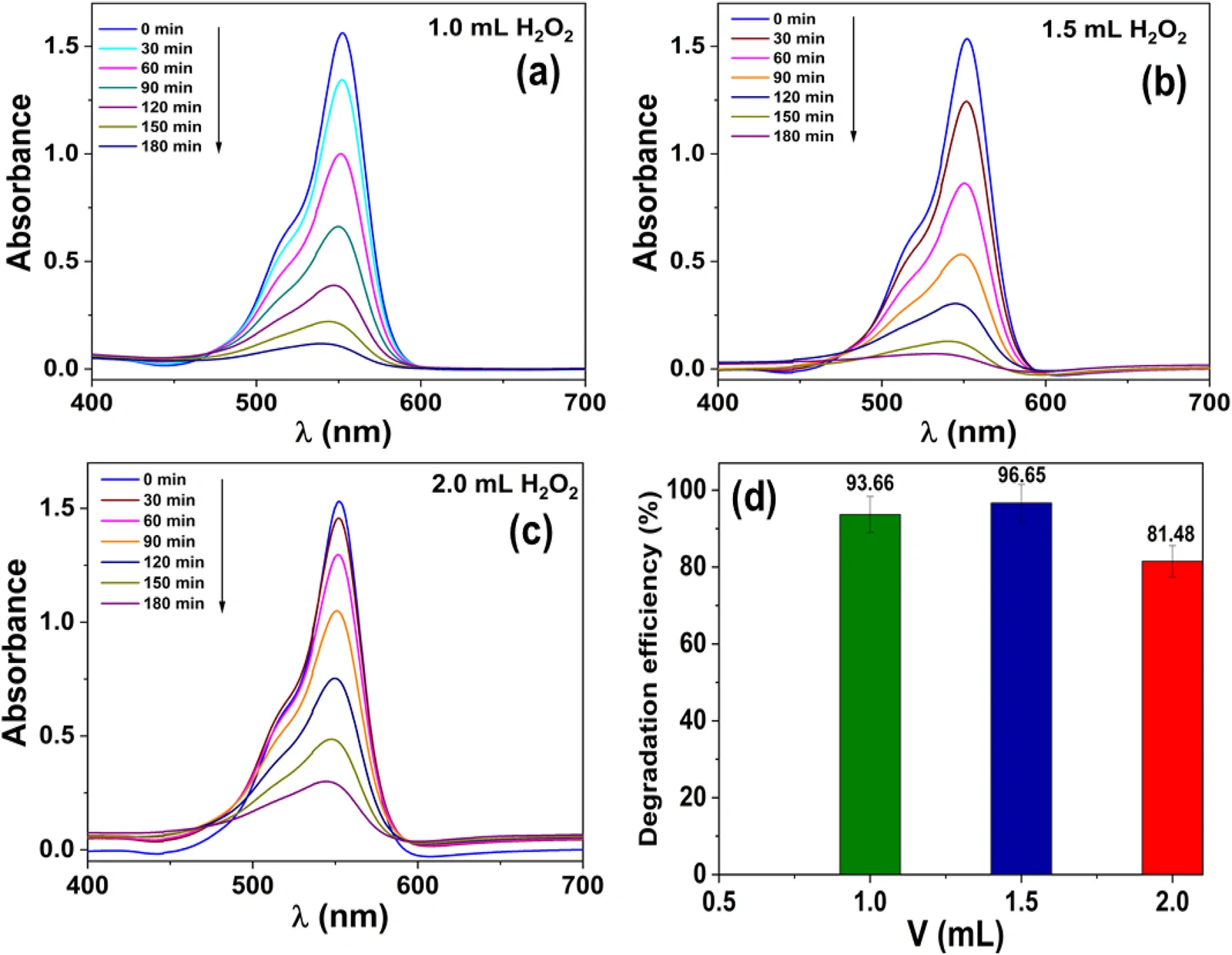
UV-vis spectral evolution of RhB during visible-light photo-Fenton degradation in the presence of Cu_0_._4_Co_0_._6_Fe_2_O_4_ and different H_2_O_2_ dosages: (a) 1.0 mL, (b) 1.5 mL, and (c) 2.0 mL of 30% H_2_O_2_; (d) corresponding RhB degradation efficiencies after 240 min of irradiation.

Under all investigated conditions, the characteristic RhB absorption band centred at approximately 554 nm decreases progressively with irradiation time. The extent of absorbance loss varies markedly with H_2_O_2_ dosage, indicating a pronounced dependence of catalytic performance on oxidant dosage within the investigated operating range.

Increasing the volume of 30 wt% H_2_O_2_ from 1.0 to 1.5 mL increases RhB degradation from 93.66% to 96.65% after 240 min of visible-light irradiation. A further increase to 2.0 mL reduces the degradation efficiency to 81.48%. Relative to the highest degradation efficiency achieved under the investigated conditions, increasing the H_2_O_2_ dosage to 2.0 mL lowers RhB removal by 15.17 percentage points. These observations identify 1.5 mL of 30 wt% H_2_O_2_ as the optimum dosage under the adopted experimental conditions.

Under all investigated conditions, the characteristic RhB absorption band centered at approximately 554 nm gradually decreased with irradiation time, reflecting the continuous removal of dye molecules during the reaction.

The catalytic response does not vary proportionally with oxidant dosage. Increasing the H_2_O_2_ volume from 1.0 to 1.5 mL improves RhB degradation by 2.99 percentage points, whereas the same dosage increment from 1.5 to 2.0 mL produces a decrease of 15.17 percentage points. The markedly larger decline observed at the highest dosage indicates that increasing H_2_O_2_ beyond the optimum dosage does not lead to further improvement in catalytic performance. The present dataset identifies an optimum dosage but does not establish the mechanistic origin of the decline observed at higher H_2_O_2_ concentrations.

Accordingly, 1.5 mL of 30 wt% H_2_O_2_ was adopted for all subsequent experiments, while the mechanistic implications of oxidant dosage are discussed separately in Section 3.5.2.

#### 3.2.4. Effect of catalyst dosage

Because the optimum catalyst composition and H_2_O_2_ dosage were established in Sections 3.2.2 and 3.2.3, respectively, the influence of catalyst dosage was subsequently evaluated under these optimized conditions. The corresponding UV-vis spectral evolution and RhB degradation efficiencies are presented in Fig. 15.

**Fig. 15:**
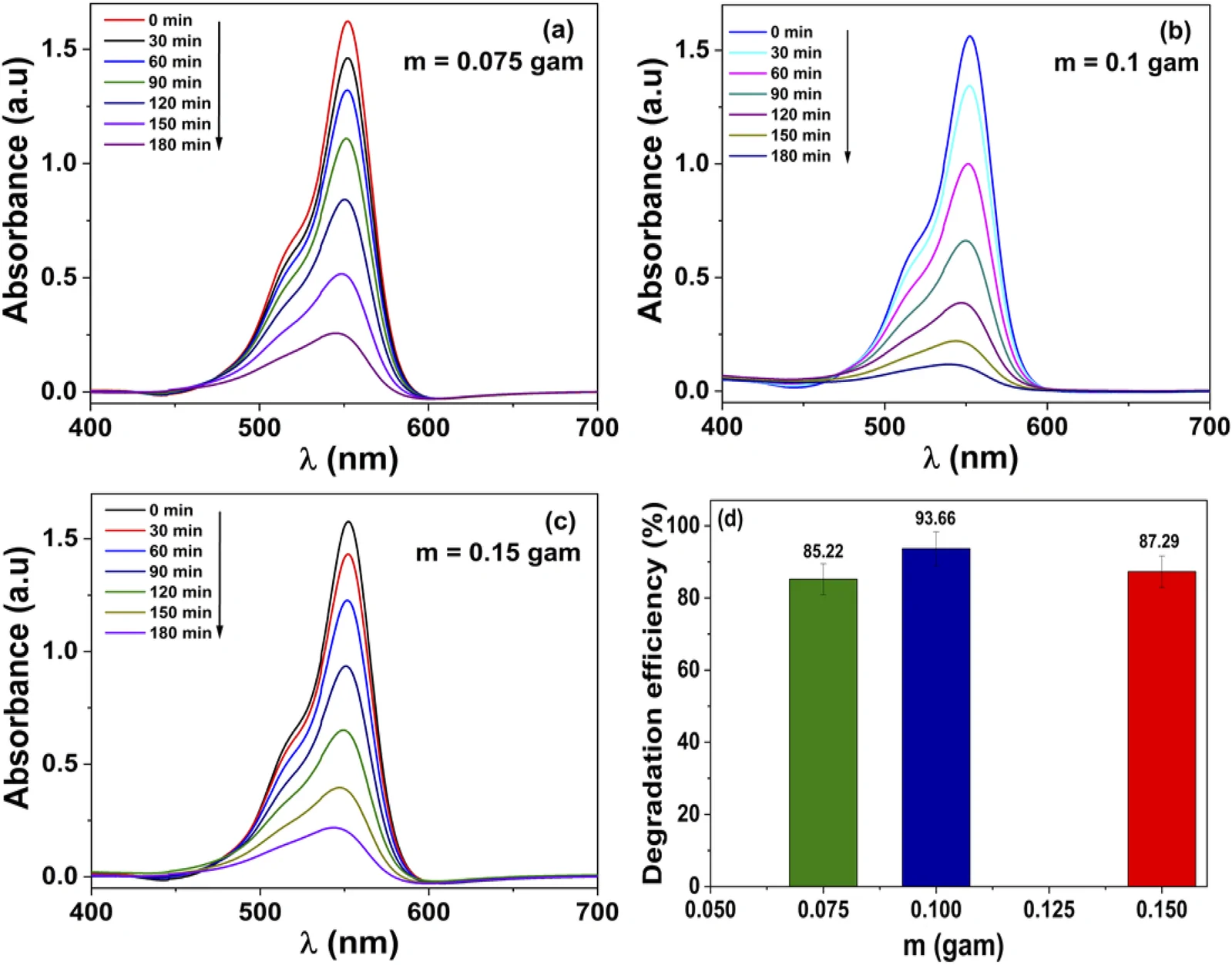
UV-vis spectral evolution of Rhodamine B (RhB) during visible-light photo-Fenton degradation in the presence of Cu_0_._4_Co_0_._6_Fe_2_O_4_ and H_2_O_2_ at catalyst dosages of (a) 0.075 g, (b) 0.10 g, and (c) 0.15 g. (d) RhB degradation efficiencies obtained after 180 min of irradiation as a function of catalyst dosage.

Under all investigated conditions, the characteristic RhB absorption band centred at approximately 554 nm decreases progressively with irradiation time. The extent of absorbance loss differs systematically with catalyst dosage, indicating a clear dependence of catalytic performance on catalyst loading within the investigated operating range.

Increasing the catalyst dosage from 0.075 to 0.10 g increases the RhB degradation efficiency from 85.22% to 93.66% after 180 min of visible-light irradiation. Further increasing the catalyst dosage to 0.15 g decreases the degradation efficiency to 87.29%, corresponding to a reduction of 6.37 percentage points relative to the highest degradation efficiency achieved under the investigated conditions. The improvement observed between 0.075 and 0.10 g is consistent with increased participation of catalytically accessible surface sites, although the present experiments do not directly quantify the number of active sites. These observations indicate that catalytic performance reaches a maximum at 0.10 g under the investigated conditions.

The catalytic response does not vary proportionally with catalyst dosage. Increasing the catalyst amount from 0.075 to 0.10 g improves RhB degradation by 8.44 percentage points, whereas the subsequent increase from 0.10 to 0.15 g decreases the degradation efficiency by 6.37 percentage points. The markedly different responses to the two dosage increments indicate that increasing catalyst loading beyond 0.10 g does not provide additional catalytic benefit under the present reaction conditions. The lower degradation efficiency observed at 0.15 g is consistent with optical shielding effects that have frequently been reported for concentrated photocatalyst suspensions, where increased particle concentrations reduce effective light penetration through the reaction medium. The present dataset, however, does not distinguish this effect from other factors that may accompany increased catalyst loading.

Accordingly, 0.10 g of Cu_0_._4_Co_0_._6_Fe_2_O_4_ was adopted for all subsequent experiments, while the mechanistic implications of catalyst dosage are integrated with the overall photo-Fenton mechanism in Section 3.5.2.

#### 3.2.5. Effect of initial rhodamine B concentration

The influence of the initial Rhodamine B (RhB) concentration was evaluated using the optimized Cu_0_._4_Co_0_._6_Fe_2_O_4_/H_2_O_2_ system established in the preceding sections. The corresponding degradation efficiencies are presented in Fig. 16. Increasing the initial RhB concentration from 5 to 15 mg L^−1^ produces a gradual decrease in degradation efficiency over the investigated concentration range.

**Fig. 16:**
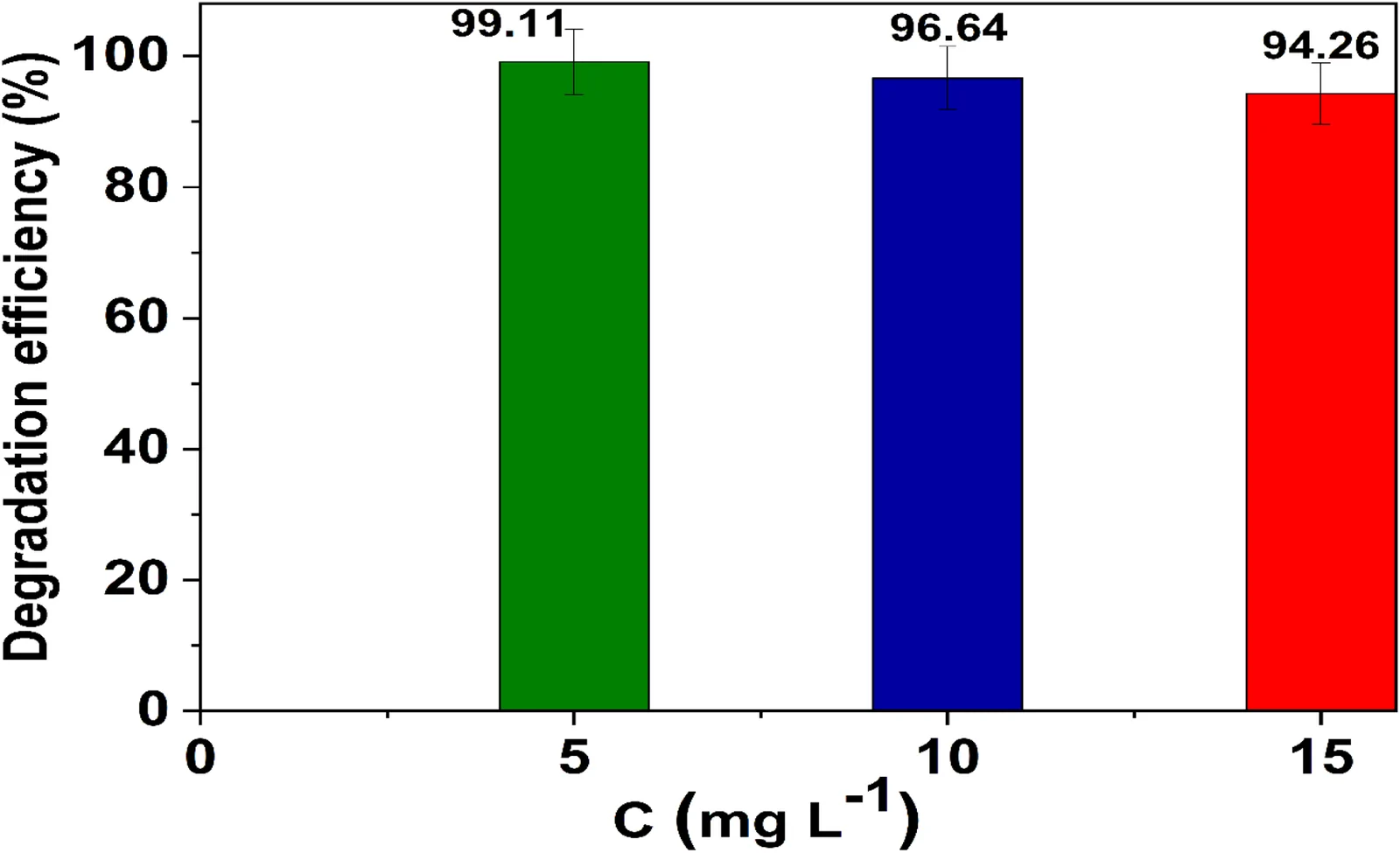
Effect of the initial rhodamine B concentration (5–15 mg L^−1^) on the visible-light photo-Fenton degradation efficiency over Cu_0_._4_Co_0_._6_Fe_2_O_4_/H_2_O_2_ after 180 min of irradiation. Error bars represent standard deviations obtained from triplicate experiments.

After 180 min of visible-light irradiation, RhB degradation decreases from 99.11% at 5 mg L^−1^ to 96.64% and 94.26% at 10 and 15 mg L^−1^, respectively. Despite a threefold increase in the initial pollutant concentration, the reduction in degradation efficiency is limited to 4.85 percentage points. This comparatively small decline indicates that the optimized photo-Fenton system maintains high degradation efficiency despite increasing pollutant loading over the investigated concentration range.

The gradual decrease in degradation efficiency is consistent with the increasing ratio between RhB molecules and the fixed quantities of catalyst and H_2_O_2_, as a larger number of dye molecules is processed without changing either the catalyst dosage or the oxidant concentration. The lower degradation efficiency observed at higher RhB concentrations is also consistent with reduced light penetration in more concentrated dye solutions, although the present experiments do not distinguish this effect from other factors associated with increasing pollutant concentration.

Although degradation efficiency decreases progressively with increasing RhB concentration, 94.26% removal is still achieved at 15 mg L^−1^, the highest concentration investigated. These results suggest that the optimized Cu_0_._4_Co_0_._6_Fe_2_O_4_/H_2_O_2_ system remains effective across the investigated concentration range, while the mechanistic implications of concentration-dependent behaviour are discussed in Section 3.5.2.

#### 3.2.6. Mineralization performance evaluated by COD and TOC

Mineralization under the optimized visible-light photo-Fenton conditions was evaluated using COD and TOC measurements (Fig. 17a and b). COD decreased from approximately 490 mg L^−1^ before irradiation to about 45 mg L^−1^ after 360 min, corresponding to a COD-based mineralization efficiency of 90.8%. TOC removal increased progressively from 27.51% at 60 min to 47.65%, 62.41%, and 84.56% at 120, 180, and 240 min, respectively (Fig. 17b). The progressive increase in TOC removal provides complementary evidence of continued organic-carbon removal during irradiation and is consistent with substantial mineralization.

**Fig. 17:**
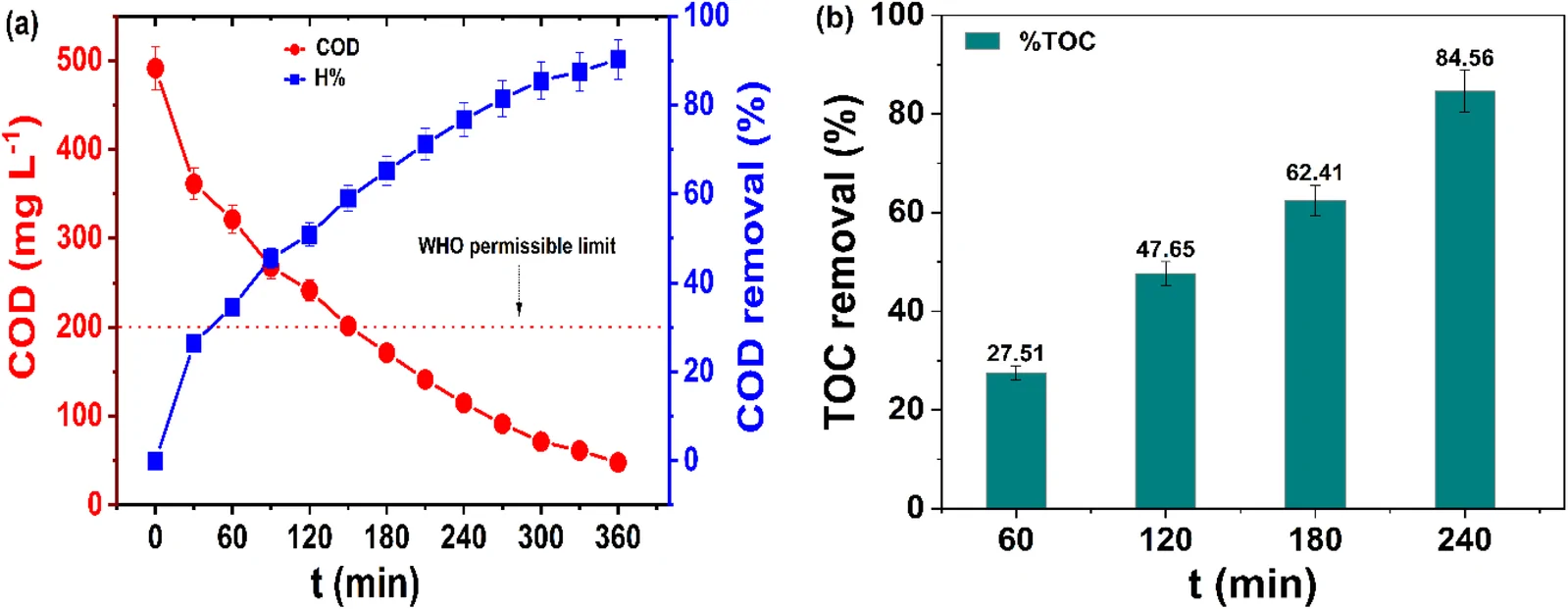
Mineralization of rhodamine B by Cu_0_._4_Co_0_._6_Fe_2_O_4_ under visible-light irradiation: (a) temporal evolution of COD concentration and COD removal efficiency, and (b) TOC removal efficiency at 60, 120, 180, and 240 min.

The near-complete RhB degradation observed under the optimized conditions, together with the lower COD- and TOC-based mineralization extents, indicates that disappearance of the parent dye does not necessarily correspond to complete removal of organic carbon. The continued decrease in COD and increase in TOC removal with irradiation time are consistent with further oxidation of soluble organic species formed during RhB degradation. However, these measurements do not identify the molecular composition or transformation pathways of the remaining intermediates. Accordingly, the combined COD and TOC results support extensive, although incomplete, mineralization under the investigated conditions.

#### 3.2.7. Reusability and structural stability

The reusability of the optimized Cu_0_._4_Co_0_._6_Fe_2_O_4_ catalyst was evaluated over four consecutive RhB degradation cycles under identical visible-light photo-Fenton conditions. The corresponding degradation profiles and final removal efficiencies are presented in Fig. 18.

**Fig. 18:**
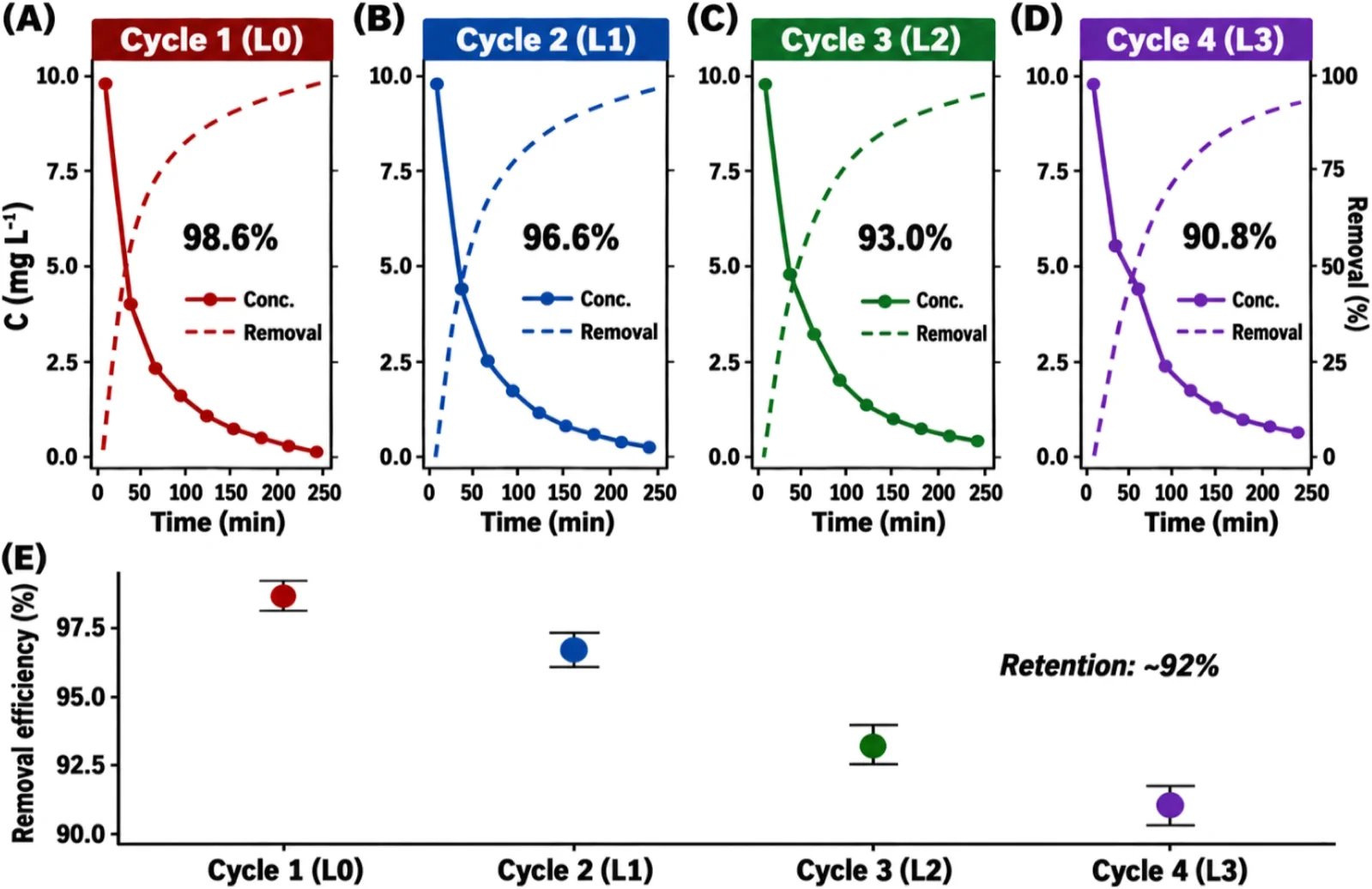
Reusability of the Cu_0_._4_Co_0_._6_Fe_2_O_4_ catalyst during visible-light photo-Fenton degradation of Rhodamine B (RhB). (A–D) Temporal evolution of RhB concentration (solid lines, left axis) and degradation efficiency (dashed lines, right axis) during four consecutive reuse cycles (L0–L3) under identical experimental conditions. (E) RhB degradation efficiency after 240 min of irradiation for each reuse cycle. Error bars represent the standard deviation of replicate measurements (*n* = 3). Approximately 92% of the initial degradation efficiency was retained after four consecutive cycles.

RhB degradation efficiency decreases gradually from 98.6% in the first cycle (L0) to 96.6%, 93.0%, and 90.8% in the second (L1), third (L2), and fourth (L3) cycles, respectively (Fig. 18A–E). The cumulative decline of 7.8 percentage points corresponds to retention of approximately 92% of the initial degradation efficiency after four consecutive reuse cycles. Despite this gradual decline, RhB removal remained above 90% throughout the four cycles, indicating retention of substantial catalytic performance under the investigated reuse conditions.

The degradation profiles remained broadly similar throughout the four reuse cycles. In each cycle, rapid RhB removal during the initial stage was followed by a slower approach to the final degradation level, while repeated reuse gradually decreased the overall degradation efficiency without substantially altering the shape of the degradation curves. This behaviour is consistent with a progressive loss of catalytic activity rather than abrupt catalyst deactivation. The origin of this activity loss, however, cannot be established from the present dataset. Gradual accumulation of surface-associated reaction intermediates or partial loss of catalytically accessible surface sites could be consistent with the observed behaviour, but the present experiments do not distinguish between these possibilities.

To further assess the structural stability of the catalyst during repeated use, the fresh Cu_0_._4_Co_0_._6_Fe_2_O_4_ catalyst and the catalyst recovered after four reuse cycles were characterized by XRD and FTIR (Fig. 19).

**Fig. 19:**
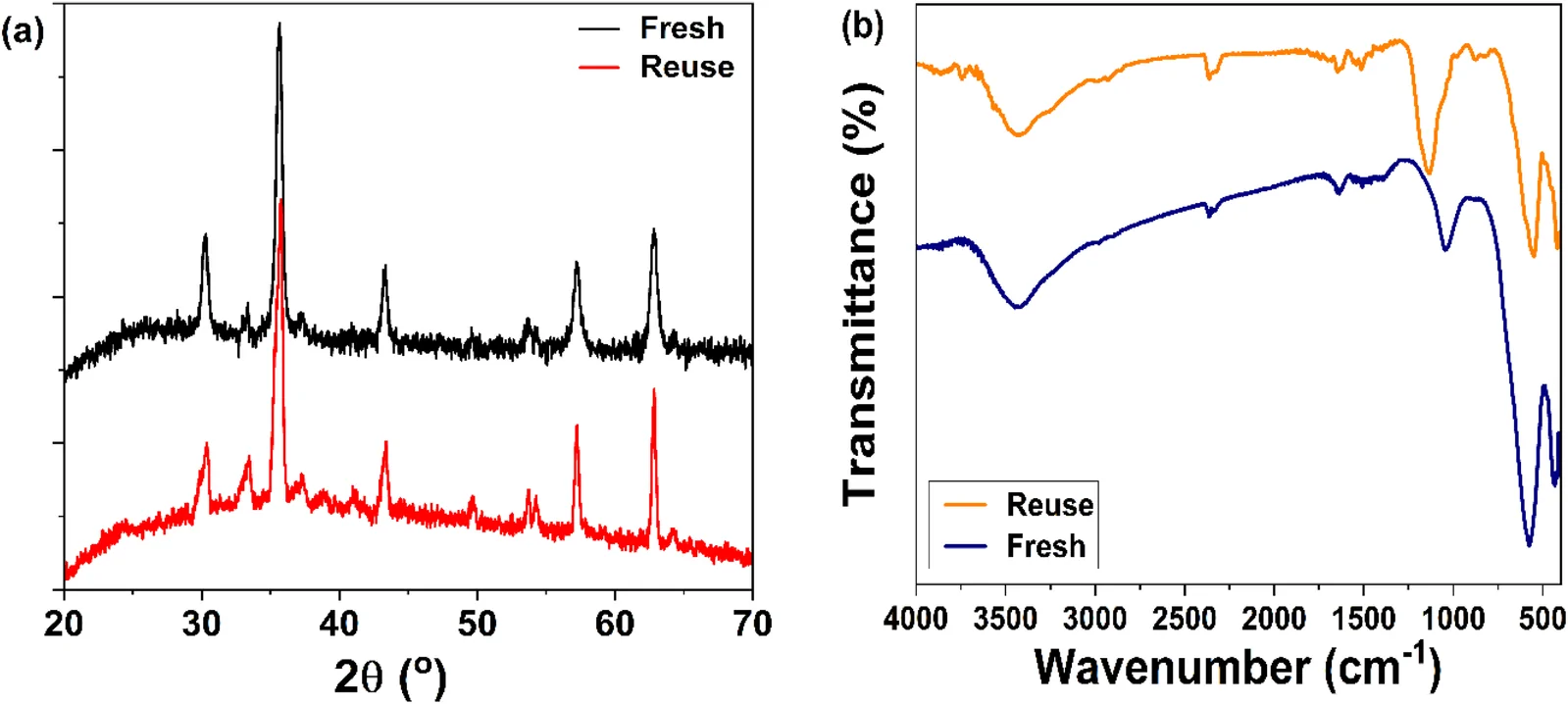
Comparison of the (a) XRD patterns and (b) FTIR spectra of fresh and four-cycle-reused Cu_0_._4_Co_0_._6_Fe_2_O_4_.

The XRD patterns of the fresh and reused catalysts retain the characteristic diffraction features of the spinel ferrite phase, with no apparent new crystalline reflections after four catalytic cycles. The FTIR spectra likewise retain the characteristic metal–oxygen vibrational bands, consistent with retention of the fundamental vibrational features of the ferrite framework after reuse.

Together with the retention of approximately 92% of the initial catalytic activity, these results support preservation of the spinel framework and characteristic metal–oxygen vibrational features under the investigated reuse conditions. Nevertheless, these analyses do not establish complete invariance of surface morphology or oxidation states. Accordingly, the present characterization is interpreted as evidence for preservation of the crystalline framework and principal metal–oxygen vibrational features rather than as definitive proof of complete structural and surface-chemical invariance after repeated use.

The observed activity retention, together with the preservation of the principal XRD and FTIR features, indicates that the Cu_0_._4_Co_0_._6_Fe_2_O_4_ catalyst maintains substantial structural and catalytic integrity over four consecutive reuse cycles under the investigated conditions. The influence of catalyst reuse on reaction kinetics is examined in the following section.

### 3.3. Kinetic analysis

To evaluate the degradation kinetics and assess the relative suitability of empirical kinetic descriptions, the experimental data were analysed using zero-order, pseudo-first-order (PFO), and pseudo-second-order (PSO) models. The PFO kinetic analysis was performed over the 30–210 min irradiation interval, with the 240 min data point excluded from kinetic fitting and used only to report the final degradation efficiency. The corresponding kinetic plots are presented in Fig. 20, and detailed PFO regression statistics are provided in Table S5 (SI).

**Fig. 20:**
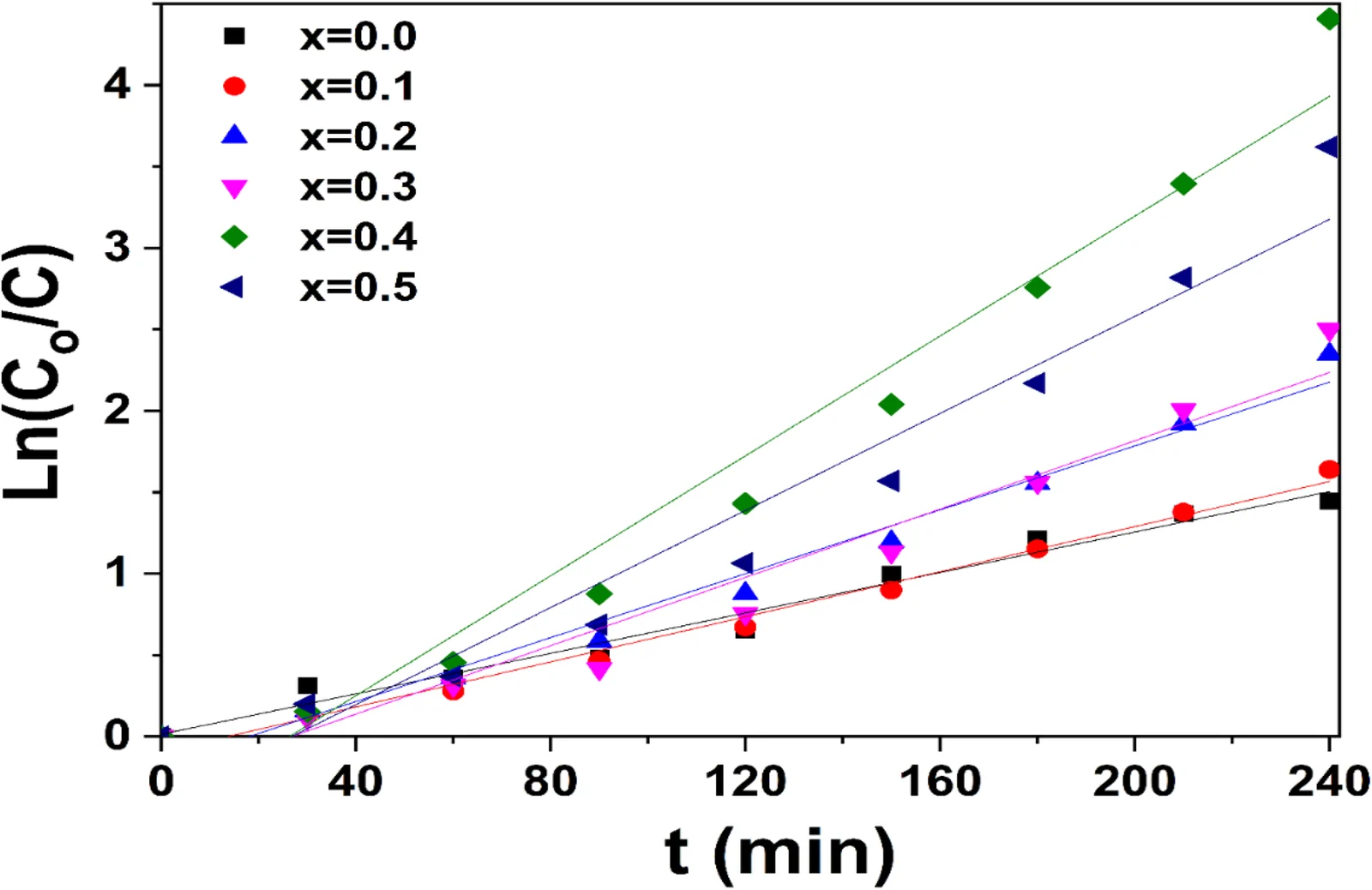
Pseudo-first-order kinetic for RhB degradation over Cu_*x*_Co_1−*x*_Fe_2_O_4_ (*x* = 0–0.5) in the presence of H_2_O_2_ under visible-light irradiation.

Linear regression of ln(*C*_0_/*C*_*t*_) *versus* irradiation time over 30–210 min yielded *R*^2^ values of 0.960–0.993 across the six catalyst compositions. The apparent rate constant increased from 0.642 × 10^−2^ min^−1^ for pristine CoFe_2_O_4_ to 1.844 × 10^−2^ min^−1^ for Cu_0_._4_Co_0_._6_Fe_2_O_4_, followed by a decrease to 1.469 × 10^−2^ min^−1^ at *x* = 0.5. Thus, the apparent kinetic response varies non-monotonically with Cu substitution, with the highest apparent rate at *x* = 0.4.

To examine whether PFO provides a consistent empirical description across the catalyst series, the experimental data were subsequently compared using zero-order, PFO, and PSO models by nonlinear least-squares fitting of *C*_*t*_/*C*_0_ over the same 30–210 min irradiation interval. Model performance was evaluated using *R*^2^, adjusted *R*^2^, RMSE, SSE, AICc, and ΔAICc, with the corresponding model parameters and statistical indicators summarized in Table S6 (SI). The comparison indicates composition-dependent model suitability. The zero-order model is favoured by AICc for *x* = 0, 0.3, and 0.5, whereas the PFO model is favoured for *x* = 0.1, 0.2, and 0.4. The PSO model is not favoured for any composition. Thus, no single empirical kinetic model provides the most appropriate description across the complete Cu_*x*_Co_1−*x*_Fe_2_O_4_ series. The PFO model is therefore not interpreted as a mechanistic assignment of reaction order.

Nevertheless, the PFO model was retained as a standardized empirical descriptor for comparing apparent degradation rates across the catalyst series. The kinetic trend is consistent with the degradation efficiencies discussed in Section 3.2.2: Cu_0_._4_Co_0_._6_Fe_2_O_4_ exhibits both the highest apparent rate constant and the highest RhB removal, whereas further Cu substitution to *x* = 0.5 decreases both parameters. Notably, the lowest optical band gap occurs at *x* = 0.1 rather than at the composition showing the highest degradation rate. The kinetic response is therefore not readily attributable to optical band-gap variation alone. More broadly, the composition-dependent activity appears to reflect the combined influence of multiple physicochemical characteristics, and their interplay is discussed further in Section 3.5.1.

Minor deviations from linearity are observed at later irradiation times, particularly for the more active catalysts. Accordingly, the *k*_1_ values are treated as apparent comparative rate constants, rather than intrinsic mechanistic rate constants or evidence of a specific reaction order.

### 3.4. Comparison with previously reported photocatalysts

To contextualize the performance of the optimized Cu_0_._4_Co_0_._6_Fe_2_O_4_ catalyst, its RhB degradation performance was compared with representative ferrite-based and non-ferrite photocatalysts reported under visible-light irradiation (Table 5). Because photocatalytic performance depends strongly on irradiation source, catalyst dosage, pollutant concentration, oxidant dosage, reaction time, reactor configuration, and solution chemistry, the comparison is intended as a qualitative benchmark rather than a direct ranking of catalytic performance.

**Table 5:** Benchmark comparison of the present catalyst with representative photocatalysts for rhodamine B degradation under visible-light irradiation^a^

Catalyst	Light	RhB (mg L^−1^)	Dose (g L^−1^)	Time (min)	Removal (%)	*k* (min^−1^)	Strategy	Ref.
**A. Ferrite-based photocatalysts**								
CoFe_2_O_4_/g-C_3_N_4_	Halogen (500 W)	—	0.60	120	57.0	—	Z-scheme heterojunction	30
Mg_0_._4_Zn_0_._1_Ca_0_._5_Fe_2_O_4_	UV (300 W)	5	0.05	80	99.5	0.130	Ca doping	31
Zn_0_._5_Ni_0_._3_Cu_0_._2_Fe_1_._9_Gd_0_._1_O_4_	Visible (300 W)	10	5.00	100	99.84	—	Gd doping	32
Cu_0_._6_Ni_0_._4_Fe_2_O_4_ (CNF4)	Metal-halide (150 W)	9	0.31	285	99.6	0.0141	Ni doping	33
Cu_0_._5_Ni_0_._5_Fe_2_O_4_	Visible light	7.5	1.00	120	97.25	0.024	Cu substitution	19
CoNd_0_._05_Fe_1_._95_O_4_	White LED (30 W)	10	0.75	180	94.7	0.0053	Nd doping	34
**Cu**_**0**_ **.**_**4**_ **Co**_**0**_ **.**_**6**_ **Fe**_**2**_ **O**_**4**_ **(this work)**	**White LED (30 W)**	**10**	**1.00**	**180**	**98.78**	**0.0184**	**Composition engineering**	**This work**

**B. Representative non-ferrite photocatalysts**								
MWCN (WO_3_/g-C_3_N_4_)	Xe (500 W)	10	1.00	50	95.5	0.063	Z-scheme	35
TiO_2_/rGO (5%)	Xe (100 W)	15	1.20	120	94.55	0.024	rGO support	36
5CuH/Bent-2	White LED (30 W)	50	0.80	60	93.6	0.016	Clay–LDH	37
CdS nanorods	White light (300 W)	10	0.125	120	88.4	—	Solvothermal	38
Ni–CdS	CFL (70 W)	10	0.50	120	95.0	0.0233	Ni doping	38 and 39

a Direct comparison among different studies should be interpreted with caution because photocatalytic performance is strongly influenced by the irradiation source, catalyst dosage, pollutant concentration, oxidant dosage, reactor configuration, and solution chemistry. Therefore, the present comparison is intended to provide a qualitative benchmark rather than a direct ranking of catalytic performance.

Within the ferrite family (Table 5A), Cu_0_._4_Co_0_._6_Fe_2_O_4_ achieved 98.78% RhB removal within 180 min under 30 W white LED irradiation, with an apparent rate constant of 0.0184 min^−1^. Comparable degradation efficiencies have been reported for Ca-doped Mg–Zn ferrite (99.5%) and Gd-doped Ni–Cu–Zn ferrite (99.84%).^31,32^ Ni-substituted Cu ferrite also achieved 99.6% RhB removal, although over a longer irradiation period of 285 min.^33^ Cu_0_._5_Ni_0_._5_Fe_2_O_4_ showed 97.25% removal within 120 min under visible-light irradiation.^19^ These systems were nevertheless evaluated under different irradiation sources, catalyst dosages, and reaction conditions, limiting direct comparison of their apparent activities with the present catalyst. Under the same nominal illumination power and reaction time (30 W white LED, 180 min), Nd-doped Co ferrite achieved 94.7% RhB removal,^34^ whereas CoFe_2_O_4_/g-C_3_N_4_ achieved 57.0% under a 500 W halogen lamp.^30^ Overall, the present catalyst achieves RhB removal within the range reported for ferrite-based photocatalysts, although differences in experimental conditions preclude direct performance ranking.

Comparison with representative non-ferrite photocatalysts (Table 5B) leads to a similar conclusion. The WO_3_/g-C_3_N_4_ Z-scheme achieved an apparent rate constant of 0.063 min^−1^, higher than that of the present catalyst, but was evaluated under a 500 W Xe lamp.^35^ TiO_2_/rGO and Cu–Mg–Al LDH/bentonite achieved comparable RhB removal using composite or supported architectures,^36,37^ while CdS nanorods and Ni-doped CdS provided further examples of semiconductor-based and doped photocatalysts.^38,39^ Thus, apparent rate constants cannot be interpreted independently of irradiation conditions, catalyst architecture, and reaction configuration.

The literature comparison also highlights a distinction in materials-design strategy. Several reported systems employ heterojunction formation, conductive supports, rare-earth incorporation, or multicomponent architectures to modify photocatalytic performance.^30,32,36–39^ In contrast, the present study examines Cu/Co composition engineering within a single-phase spinel ferrite framework, with the highest RhB removal observed for Cu_0_._4_Co_0_._6_Fe_2_O_4_. This approach modifies composition without introducing a secondary functional phase, providing a comparatively simple basis for probing composition-dependent photo-Fenton activity.

When considered together with the structural, optical, and kinetic results in Sections 3.1–3.3, the literature comparison further indicates that catalytic activity is not readily attributable to any single physicochemical descriptor examined in this study. The optimum Cu_0_._4_Co_0_._6_Fe_2_O_4_ composition coincides with coordinated changes in structural, textural, optical, and kinetic characteristics rather than with the maximum or minimum of any single measured descriptor. The comparison therefore provides external context for the composition-dependent activity observed here, while avoiding direct attribution of catalytic performance to an individual material property. The underlying composition–structure–activity relationship is discussed further in Section 3.5.1.

### 3.5. Mechanistic insights into visible-light photo-Fenton degradation of RhB

#### 3.5.1. Composition–structure–activity relationships

To quantitatively assess the composition–structure–activity relationship, Spearman rank correlations were calculated between *k*_app_ and the physicochemical descriptors measured across the complete Cu_*x*_Co_1−*x*_Fe_2_O_4_ series (*x* = 0–0.5, *n* = 6; Table S7 (SI) and Fig. 21).

**Fig. 21:**
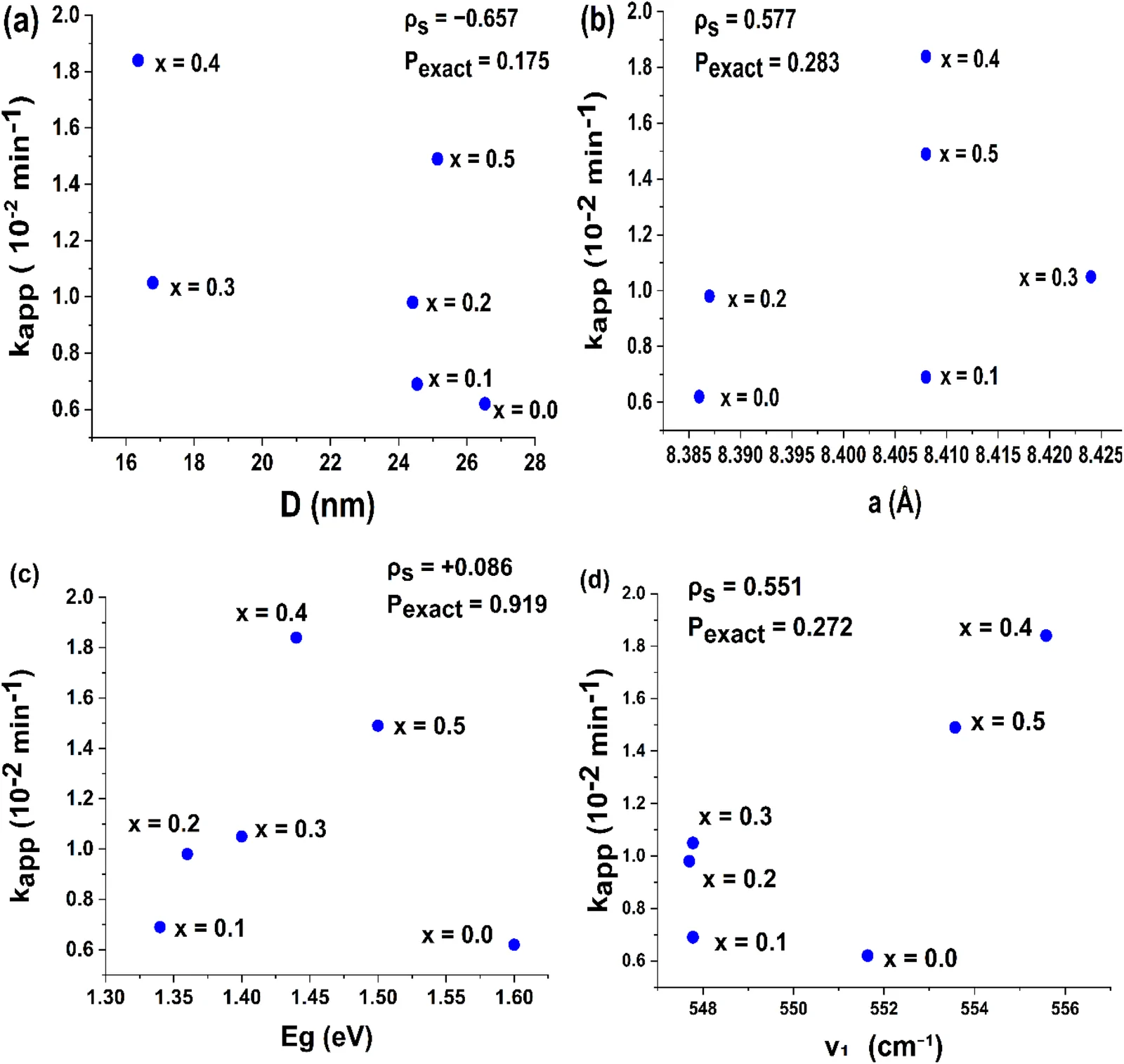
Quantitative assessment of composition–structure–activity relationships in Cu_*x*_Co_1−*x*_Fe_2_O_4_. (a–d) Spearman rank correlations between the apparent rate constant (*k*_app_) and crystallite size (*D*), lattice parameter (*a*), optical band-gap energy (*E*_g_), and FTIR *ν*_1_ band position, respectively, across the Cu_*x*_Co_1−*x*_Fe_2_O_4_ series (*x* = 0–0.5; *n* = 6). Each point represents one composition level, with the corresponding Cu substitution level indicated adjacent to the point. Spearman correlation coefficients (*ρ*_s_) and exact two-sided permutation *P*-values are shown in each panel.

The descriptors evaluated for the activity-focused analysis included crystallite size (*D*), lattice parameter (*a*), FTIR *ν*_1_ band position, and optical band-gap energy (*E*_g_). The corresponding *ν*_2_–*k*_app_ relationship and the complete pairwise correlation matrix are provided in Fig. S1 and S2 (SI), respectively, to document the full set of evaluated associations. Spearman correlation was selected because it evaluates monotonic association without assuming a linear relationship between the descriptor and catalytic activity. Given the limited number of independent composition levels, two-sided exact permutation tests were used to assess statistical significance.

The Spearman coefficients were −0.657 for *D*, +0.577 for *a*, +0.551 for *ν*_1_, −0.147 for *ν*_2_, and +0.086 for *E*_g_, with exact *P*-values of 0.175, 0.283, 0.272, 0.778, and 0.919, respectively (Table S8 and Fig. S1, S2 (SI)). None of the descriptors showed a statistically significant monotonic association with *k*_app_. Moreover, the minimum *E*_g_ was observed at *x* = 0.1, whereas the maximum *k*_app_ occurred at *x* = 0.4. Thus, the observed activity maximum is not reproduced by the band-gap trend alone. The quantitative results are consistent with the broader composition-dependent structural changes observed experimentally. From *x* = 0 to *x* = 0.4, the Cu-containing series exhibited progressive crystallite refinement, with *D* decreasing from 26.53 to 16.36 nm and diffraction-line broadening increasing from 0.311° to 0.504°. The corresponding increase in calculated dislocation density provides a complementary description of the same crystallite refinement rather than an independent structural variable. The FTIR *ν*_2_ band shifted from 424.34 to approximately 474.49 cm^−1^, while TEM measurements showed a reduction in average particle size from approximately 30 to 20 nm between the pristine CoFe_2_O_4_ and the *x* = 0.4 material. These observations are consistent with composition-dependent changes in the average ferrite crystal framework and metal–oxygen vibrational characteristics across the Cu-substituted series.

The textural and optical characteristics did not follow the same trend as catalytic activity. BET surface area decreased from 7.55 to 3.60 m^2^ g^−1^ between *x* = 0 and *x* = 0.4, corresponding to a decrease of approximately 52%, whereas the apparent rate constant increased from 0.62 × 10^−2^ to 1.84 × 10^−2^ min^−1^ over the same compositional interval. This contrast suggests that BET surface area alone is not an adequate predictor of photo-Fenton activity in the present system. The minimum *E*_g_ of 1.34 eV occurred at *x* = 0.1, further showing that neither textural surface area nor optical band-gap energy alone reproduces the activity trend. BET analysis quantifies the N_2_-accessible surface area but does not directly resolve the nature or accessibility of the sites involved in the photo-Fenton process. Thus, the activity enhancement coincides with multiple composition-dependent physicochemical changes rather than with an increase in BET surface area alone. At *x* = 0.5, the decrease in activity coincided with partial recovery of crystallite size, a decrease in calculated dislocation density, and widening of the optical band gap. Thus, neither the structural, textural, nor optical descriptors examined here exhibits a simple one-parameter trend that reproduces the catalytic activity across the full composition series.

Together, the correlation analysis and the complementary characterization results do not support attributing the enhanced activity at *x* = 0.4 to any single measured structural or optical descriptor. The results instead point to composition-dependent changes across multiple physicochemical characteristics. The individual causal contributions of these characteristics therefore remain unresolved within the present six-composition dataset.

Recent literature further highlights the importance of compositional and structural control in ferrite-based and Cu-containing functional materials. Targeted compositional modification has been shown to influence the microstructure and functional properties of MnZn ferrites,^40^ while recent Cu-containing catalytic systems illustrate how the Cu chemical environment and catalyst architecture can influence catalytic behaviour.^41,42^ These studies provide updated context for the composition–structure–activity relationships investigated in the present Cu_*x*_Co_1−*x*_Fe_2_O_4_ system.

Because dislocation density (*δ*) was calculated directly from crystallite size (*δ* = 1/*D*^2^), it was not treated as an independent descriptor in the correlation analysis. BET surface area and TEM-derived particle size were also excluded from the correlation analysis because these measurements were available only for *x* = 0 and *x* = 0.4. With only two composition-level observations, these variables provide no meaningful basis for rank correlation across the complete composition series and are therefore retained as complementary characterization results.

#### 3.5.2. Active-species analysis and proposed degradation pathway

Reactive-species trapping experiments were performed to identify the oxidative species participating in RhB degradation over the optimized Cu_0_._4_Co_0_._6_Fe_2_O_4_/H_2_O_2_/visible-light system (Fig. 22). IPA, benzoquinone (BQ), and EDTA were employed as selective scavengers for ˙OH, 
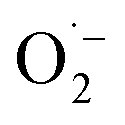
, and h^+^, respectively. Suppression of RhB degradation by all three scavengers demonstrates that multiple oxidative species contribute to the overall photo-Fenton process under the investigated conditions.

**Fig. 22:**
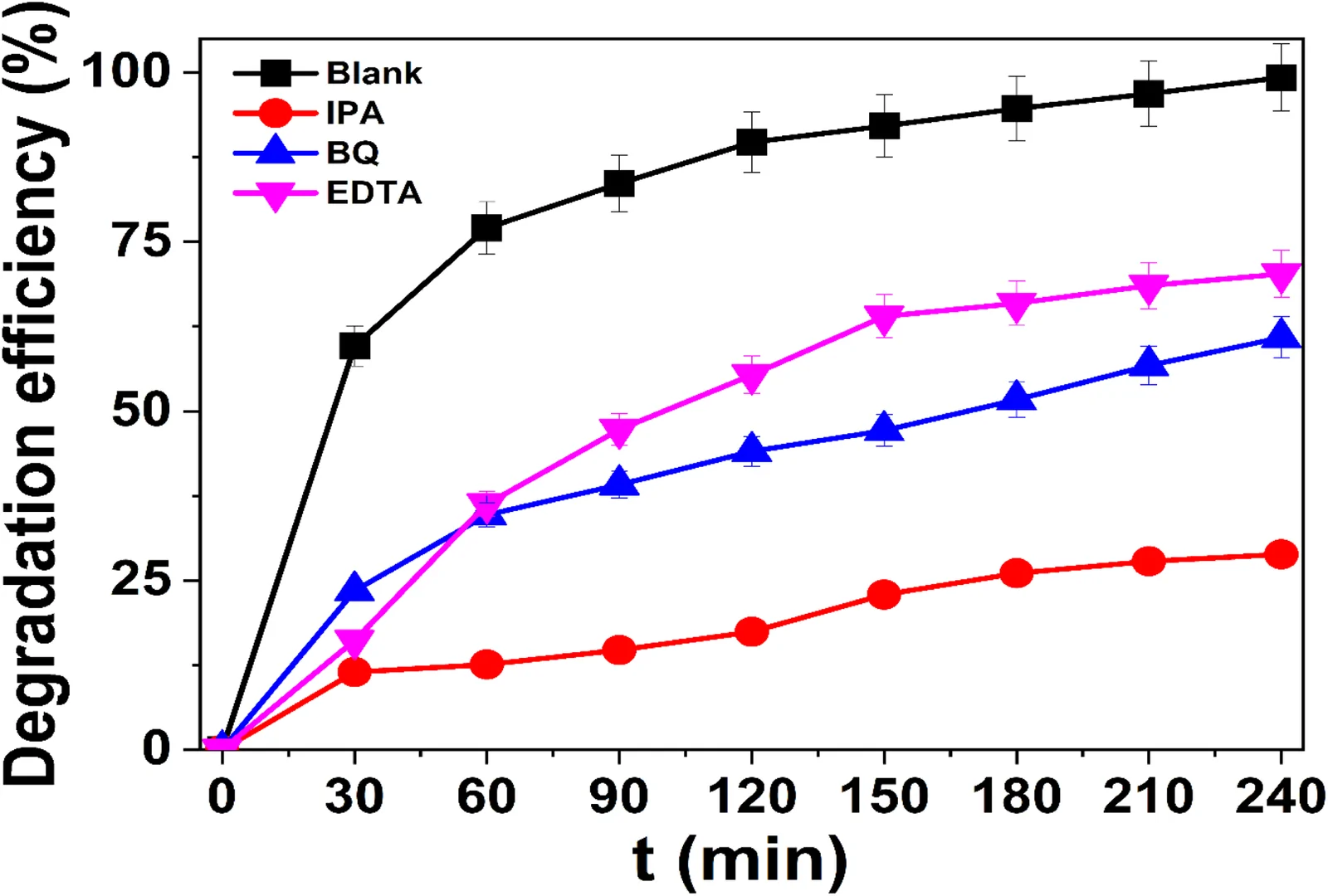
Reactive-species trapping experiments.

The strongest inhibition occurred in the presence of IPA, where RhB degradation decreased from approximately 99.1% to 28.9% after 240 min of irradiation, corresponding to a reduction exceeding 70 percentage points. By comparison, degradation decreased to 60.8% with BQ and 70.1% with EDTA. The inhibition sequence (IPA > BQ > EDTA) supports a dominant contribution from ˙OH, while 
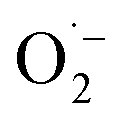
 and photogenerated holes provide secondary contributions to the overall oxidation process. Because radical-scavenging experiments cannot resolve radical concentrations or distinguish overlapping reaction routes, this ranking should be interpreted qualitatively rather than quantitatively.^43^

In heterogeneous photo-Fenton systems, H_2_O_2_ activation can involve redox processes associated with surface-accessible Fe sites within the iron-containing spinel ferrite, without requiring a freely dissolved Fe^2+^/Fe^3+^ pool in the bulk solution. In the present Cu_0_._4_Co_0_._6_Fe_2_O_4_ catalyst, XPS shows Fe in an oxide environment; however, XPS does not by itself resolve the Fe^2+^/Fe^3+^ ratio or establish a specific Fe redox cycle under the reaction conditions. Therefore, the Fe-mediated H_2_O_2_ activation illustrated in Fig. 23 is presented as a proposed surface-associated heterogeneous process at the ferrite–solution interface rather than as evidence for a homogeneous dissolved Fe^2+^/Fe^3+^ Fenton cycle. Because dissolved Fe concentrations were not measured during the reaction, a minor contribution from Fe leaching and a corresponding homogeneous pathway cannot be completely excluded.

**Fig. 23:**
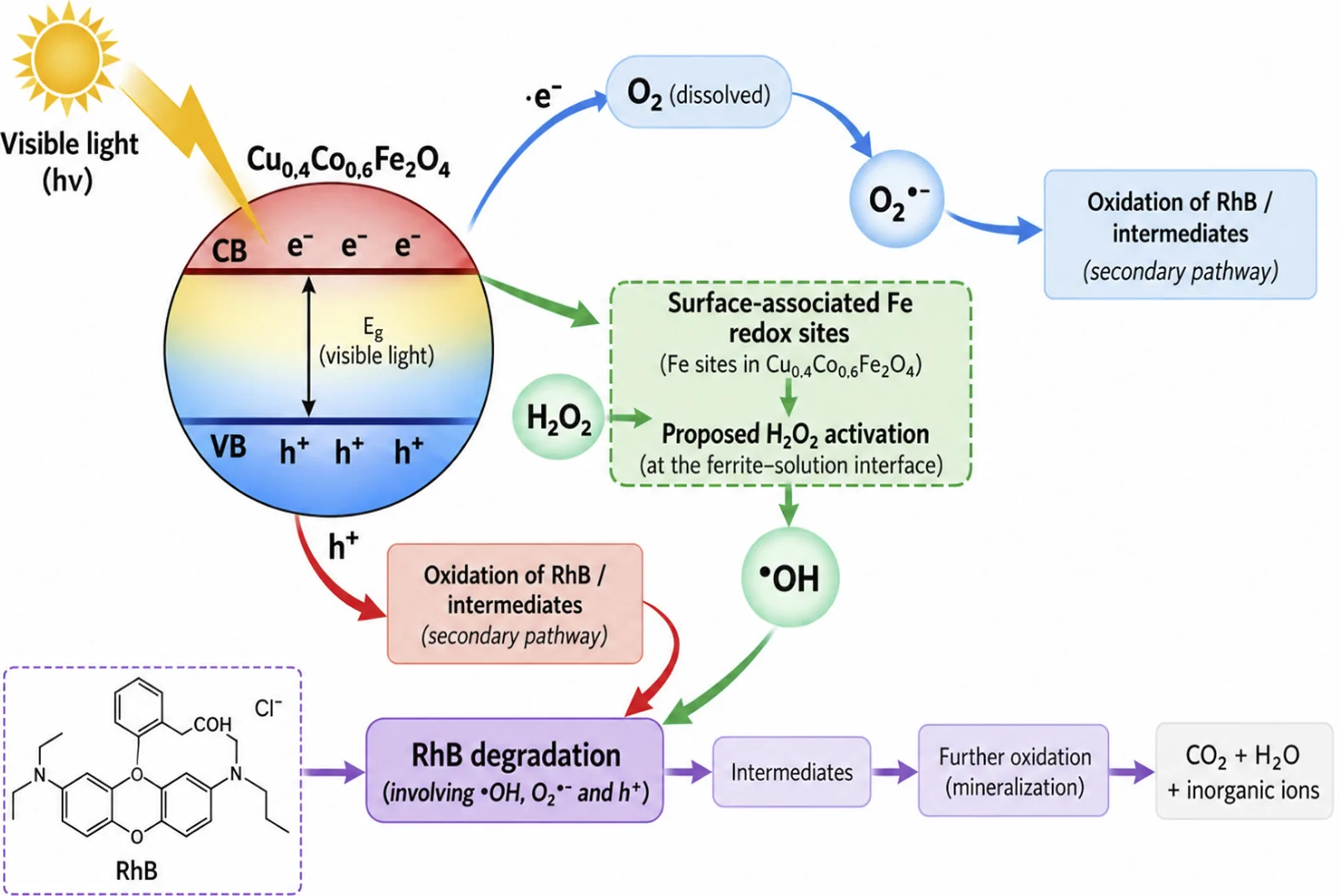
Proposed visible-light photo-Fenton degradation pathway over Cu_0_._4_Co_0_._6_Fe_2_O_4_, highlighting surface-associated Fe-mediated H_2_O_2_ activation and the inferred contributions of ˙OH, 
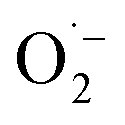
, and h^+^.

The trapping experiments are consistent with the mineralization behaviour discussed in Section 3.2.6. Under the optimized conditions, 98.78% RhB removal was accompanied by substantial COD and TOC removal, indicating that oxidation extends beyond disappearance of the parent dye. Although the molecular identities of the degradation intermediates were not determined because LC-MS or GC-MS analysis was not performed, the observed degradation and mineralization behaviour is consistent with a sequential oxidative process. The initial disruption of the RhB chromophoric structure may be followed by further oxidation of residual aromatic and other soluble organic species, accompanied by progressive mineralization. The continued decrease in COD and increase in TOC removal with irradiation time support continued oxidation of residual organic matter. However, these bulk measurements do not resolve the molecular identity or transformation sequence of individual intermediates. Accordingly, the proposed degradation sequence is regarded as a chemically plausible interpretation rather than direct experimental identification.

Fig. 23 summarizes a proposed visible-light photo-Fenton pathway consistent with the experimental observations and established heterogeneous Fenton chemistry.^44–46^ Within this framework, hydroxyl radicals are inferred to provide the principal oxidation route, whereas superoxide radicals and photogenerated holes make secondary contributions to RhB degradation. The collected evidence does not distinguish the relative importance of the individual radical-generation pathways, and the proposed sequence should therefore be interpreted as a chemically plausible reaction scenario rather than a verified sequence of elementary steps.

Several experimental techniques commonly used for mechanistic verification were beyond the scope of the present study. Reactive oxygen species were inferred from trapping experiments rather than directly detected by ESR/EPR, while photocurrent, electrochemical impedance spectroscopy (EIS), and post-reaction XPS measurements were not performed. Consequently, the participation of ˙OH, 
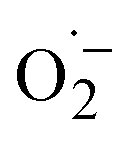
, and h^+^ is supported by the experimental observations, whereas the individual radical-generation steps and surface redox processes remain literature-supported interpretations requiring further experimental verification.

### 3.6. Environmental implications

The environmental relevance of the present study extends beyond high RhB removal. Across the Cu_*x*_Co_1−*x*_Fe_2_O_4_ series, the highest photo-Fenton activity was achieved within a narrow compositional window rather than through continuous Cu enrichment. This behaviour suggests that rational composition engineering can avoid unnecessary Cu enrichment while maintaining high catalytic activity, providing a composition-guided approach to the development of spinel ferrite photo-Fenton catalysts.

Under the optimized conditions, Cu_0_._4_Co_0_._6_Fe_2_O_4_ achieved 98.78% RhB degradation and approximately 90.8% COD removal. Because dye decolourization alone does not establish effective removal of organic matter, the substantial COD reduction indicates oxidation beyond chromophore destruction. The observed COD and TOC reductions further support the potential of the optimized composition for advanced oxidation of dye-bearing wastewater, while molecular-level identification of degradation intermediates remains necessary for a more complete assessment.

From a materials perspective, the solution-combustion route offers compositional flexibility through a comparatively straightforward synthesis process. The use of nitrate precursors and urea enables systematic control of the Cu/Co ratio while retaining the spinel ferrite framework across the investigated compositions. This provides a convenient experimental basis for composition-guided optimization of ferrite photo-Fenton catalysts without requiring increasingly complex catalyst architectures.

Several challenges remain before practical application can be assessed. The present study used a model dye under controlled laboratory conditions and therefore does not capture the complexity of authentic industrial wastewater. Competing organic and inorganic constituents, suspended solids, and variations in pH and ionic strength may affect light penetration, H_2_O_2_ utilization, and access to catalytically active sites. Long-term stability and potential Cu/Co/Fe leaching also require systematic evaluation under repeated use and continuous-flow operation. For continuous-flow treatment, catalyst retention or recovery, regeneration, hydraulic residence time, catalyst–pollutant contact, light distribution, and H_2_O_2_ dosing and delivery will require further optimization. In addition, degradation-product profiles and potential toxicity should be evaluated because high dye removal does not necessarily establish detoxification. These considerations define the principal validation requirements for assessing the catalyst under authentic wastewater and continuous-flow conditions.

Overall, the results establish a composition-dependent activity profile for Cu_*x*_Co_1−*x*_Fe_2_O_4_ and provide a basis for further evaluation of Cu_0_._4_Co_0_._6_Fe_2_O_4_ under authentic wastewater, prolonged operation, and continuous-flow conditions.

### 3.7. Limitations and future perspectives

The principal limitation of the present study concerns the physicochemical basis of the observed activity maximum at Cu_0_._4_Co_0_._6_Fe_2_O_4_. Although several structural, optical, and catalytic descriptors vary systematically with composition, the present measurements do not resolve their individual causal contributions. In particular, XRD, FTIR, and XPS cannot unambiguously establish Cu, Co, and Fe site occupancy within the spinel sublattices, while the single-peak Scherrer analysis provides only comparative diffraction-domain estimates. Together with the limited number of independent compositions, these constraints preclude assignment of a unique structural origin to the activity maximum. The observed composition–structure–activity relationships are therefore interpreted as experimentally supported associations rather than direct evidence of causality. Site-sensitive and *operando* characterization would be valuable for resolving the underlying structural and interfacial processes.

A second limitation concerns the molecular-level assessment of degradation and mineralization. Although 98.78% RhB degradation was accompanied by 90.8% COD removal and 84.56% TOC removal at 240 min, the molecular identities and potential toxicity of degradation intermediates were not determined. The combined COD and TOC results therefore indicate substantial but incomplete organic-carbon removal without resolving individual transformation pathways. LC-MS or GC-MS, combined with ecotoxicological assessment, would enable more direct evaluation of degradation-product profiles and potential detoxification.

Finally, the four-cycle reuse tests showed retention of approximately 92% of the initial activity, with preservation of the principal XRD and FTIR features; however, longer-term surface-chemical stability and metal leaching were not quantitatively established. Dissolved Fe and Cu were not measured during or after the reaction; therefore, metal leaching and a possible homogeneous contribution cannot be completely excluded. The proposed Fe-mediated H_2_O_2_ activation is consequently discussed as a surface-associated heterogeneous process rather than an exclusively heterogeneous pathway. Direct measurement of dissolved Fe and Cu would be required to further distinguish these contributions. Broader validation under diverse wastewater matrices, prolonged operation, catalyst regeneration, and continuous-flow conditions is therefore required to assess the practical robustness of the composition-dependent activity identified here.

## 4. Conclusion

Systematic Cu substitution revealed a distinct composition-dependent evolution of the physicochemical characteristics and visible-light photo-Fenton performance of spinel CoFe_2_O_4_ ferrites while preserving the cubic spinel framework throughout the investigated composition range. Rather than increasing monotonically with Cu content, the highest observed catalytic performance occurred at Cu_0_._4_Co_0_._6_Fe_2_O_4_, indicating that the activity maximum was reached at an intermediate Cu substitution level rather than through continuous Cu enrichment.

Under the optimized conditions, Cu_0_._4_Co_0_._6_Fe_2_O_4_ achieved 98.78% RhB degradation and exhibited an approximately 2.9-fold higher apparent reaction-rate constant than pristine CoFe_2_O_4_, while retaining approximately 92% of its initial degradation efficiency after four consecutive reuse cycles. Radical-scavenging experiments supported ˙OH as the predominant reactive species under the investigated conditions, although the elementary radical-generation pathways could not be resolved from the present measurements.

An important finding is that the highest photocatalytic activity did not coincide with either the maximum BET surface area or the minimum optical band-gap energy. The activity maximum occurred alongside pronounced changes in crystallographic and vibrational characteristics, but these features cannot be assigned as independent causal determinants from the present dataset. Within the limits of the present dataset, the activity maximum is therefore more consistently associated with the coupled evolution of composition-dependent physicochemical characteristics than with any single measured descriptor. These findings support composition-dependent tuning within the investigated Cu_*x*_Co_1−*x*_Fe_2_O_4_ system and provide experimentally supported insight into the composition–structure–activity relationships observed in its visible-light photo-Fenton performance.

## Author contributions

Conceptualization: T. T. L. N.; methodology: N. T. T. L. and T. H. L. N.; software: X. T. M., T. H. L. N. and T. M. V. N.; validation: V. N. V., T. T. L. N., X. T. M. and T. H. V.; data curation: T. T. L. N., T. H. V., T. H. L. N. and T. M. V. N.; writing – original draft preparation: T. X. V., T. H. V. and T. T. L. N.; writing – review and editing, V. T. X.; visualization: V. N. V., T. X. V., T. M. V. N., T. H. V. and X. T. M.

## Conflicts of interest

The authors declare no conflicts of interest.

## Supplementary Material

RA-016-D6RA06896K-s001

## Data Availability

Additional experimental data and supporting information are available from the corresponding author upon reasonable request. All data supporting the findings of this study are provided within the article and its supplementary information (SI). Supplementary information: additional elemental composition data (Table S1), detailed textural properties obtained from BET, *t*-plot, and BJH analyses (Tables S2 and S3), and experimental kinetic data used for pseudo-first-order analysis (Tables S4 and S5). These supplementary data support the characterization, kinetic analysis, and discussion presented in the main text. See DOI: https://doi.org/10.1039/d6ra06896k.
