# Supplementary material for: Composition–structure–activity relationships in CuxCo1−xFe2O4 spinel ferrites for visible-light photo-Fenton degradation of rhodamine B
Source: RSC Adv. 2026 Sep 30. Online ahead of print. doi: 10.1039/d6ra06896k (PMC13626155; doi:10.1039/d6ra06896k)
Supplement: Supplementary file 1 [file RA-OLF-D6RA06896K-s001.pdf]

Supplementary Information (SI) for RSC Advances.
This journal is © The Royal Society of Chemistry 2026
Supplementary Information (SI) for RSC Advances.
This journal is © The Royal Society of Chemistry

SUPPLEMENTARY INFORMATION

Title: Composition–structure–activity relationships in $Cu_xCo_{1-x}Fe_2O_4$ spinel ferrites for visible-light photo-Fenton degradation of Rhodamine B

Table S1. Theoretical and EDS-determined elemental compositions (wt%) of $CoFe_2O_4$ and $Cu_{0.4}Co_{0.6}Fe_2O_4$ ferrites.

Sample	Co (wt%)		Fe (wt%)		O (wt%)		Cu (wt%)	
	Theoretical	EDS	Theoretical	EDS	Theoretical	EDS	Theoretical	EDS
$CoFe_2O_4$	25.11	25.29	47.66	45.69	27.23	29.02	0.00	0.00
$Cu_{0.4}Co_{0.6}Fe_2O_4$	14.94	10.15	47.26	41.77	27.00	40.54	10.80	7.54

Note: Theoretical values were calculated from the nominal stoichiometric compositions. Experimental values were obtained from EDS analysis. Oxygen contents determined by EDS should be interpreted cautiously because of the limited accuracy of light-element quantification.

Table S2. Detailed textural parameters obtained from BET, t-plot, and BJH analyses.

Sample	BET surface area (m^2 g^{-1})	Micropore area (m^2 g^{-1})	External surface area (m^2 g^{-1})	BJH adsorption pore volume (cm^3 g^{-1})	BJH desorption pore volume (cm^3 g^{-1})	BJH adsorption pore width (nm)	BJH desorption pore width (nm)
$CoFe_2O_4$	7.55	0.90	6.65	0.0797	0.0710	50.07	41.29
$Cu_{0.4}Co_{0.6}Fe_2O_4$	3.60	0.86	2.74	0.0386	0.0371	56.04	43.82

Table S3. Relative changes in textural properties induced by Cu substitution in the $CoFe_2O_4$ spinel lattice.

Parameter	$CoFe_2O_4$	$Cu_{0.4}Co_{0.6}Fe_2O_4$	Change (%)
BET surface area (m^2 g^{-1})	7.55	3.60	−52.3
Total pore volume (cm^3 g^{-1})	0.0797	0.0386	−51.6
Average pore diameter (nm)	50.07	56.04	+11.9
Micropore area (m^2 g^{-1})	0.90	0.86	−4.4
External surface area (m^2 g^{-1})	6.65	2.74	−58.8

Table S4. Kinetic data for RhB degradation

t (min)	C_t/C_0 x=0	$\ln(C_0/C_t)$ x=0	C_t/C_0 x=0.1	$\ln(C_0/C_t)$ x=0.1	C_t/C_0 x=0.2	$\ln(C_0/C_t)$ x=0.2	C_t/C_0 x=0.3	$\ln(C_0/C_t)$ x=0.3	C_t/C_0 x=0.4	$\ln(C_0/C_t)$ x=0.4	C_t/C_0 x=0.5	$\ln(C_0/C_t)$ x=0.5
30	0.731	0.313	0.871	0.138	0.852	0.160	0.891	0.116	0.858	0.153	0.820	0.199
60	0.700	0.357	0.757	0.279	0.697	0.361	0.731	0.314	0.635	0.454	0.691	0.369
90	0.618	0.482	0.629	0.464	0.557	0.585	0.660	0.416	0.416	0.877	0.503	0.687
120	0.519	0.655	0.511	0.672	0.416	0.878	0.471	0.753	0.239	1.431	0.345	1.064
150	0.369	0.998	0.407	0.899	0.303	1.195	0.322	1.133	0.130	2.039	0.208	1.568
180	0.297	1.214	0.316	1.152	0.211	1.555	0.210	1.560	0.063	2.758	0.114	2.170
210	0.255	1.368	0.252	1.377	0.147	1.920	0.134	2.007	0.034	3.394	0.060	2.818
240	0.197	1.623	0.194	1.639	0.096	2.346	0.082	2.499	0.012	4.407	0.027	3.621

Note: C_t/C_0 was calculated as $\exp[-\ln(C_0/C_t)]$. Kinetic regression was performed using the data from 30 to 210 min. The 240-min data point was excluded from kinetic fitting and was used only for reporting the final degradation efficiency.

Table S5. Apparent pseudo-first-order rate constants (k) and corresponding regression coefficients (R^2) for RhB degradation over $Cu_xCo_{1-x}Fe_2O_4$ (x = 0–0.5) in the presence of H_2O_2 under visible-light irradiation.

Catalyst	Intercept	SE (intercept)	k_1 ($\times10^{-2}$ min^{-1})	SE(k_1) ($\times10^{-2}$ min^{-1})	95% CI of k_1 ($\times10^{-2}$ min^{-1})	t	p	R^2	Adj. R^2	SSE	RSE	n	df
$CoFe_2O_4$ (x=0)	−0.001143	0.078202	**0.6423**	0.0583	0.4924–0.7921	11.019	1.072×10^{-4}	0.96045	0.95056	0.042809	0.092530	7	5
$Cu_{0.1}Co_{0.9}Fe_2O_4$	−0.131000	0.034826	**0.7021**	0.0260	0.6354–0.7689	27.049	1.292×10^{-6}	0.99321	0.99152	0.008490	0.041207	7	5
$Cu_{0.2}Co_{0.8}Fe_2O_4$	−0.232000	0.064416	**0.9855**	0.0480	0.8621–1.1089	20.525	5.080×10^{-6}	0.98827	0.98534	0.029046	0.076218	7	5
$Cu_{0.3}Co_{0.7}Fe_2O_4$	−0.369000	0.126354	**1.0574**	0.0942	0.8153–1.2995	11.227	9.788×10^{-5}	0.96185	0.95231	0.111758	0.149504	7	5
$Cu_{0.4}Co_{0.6}Fe_2O_4$	−0.626714	0.139727	**1.8444**	0.1041	1.5767–2.1121	17.710	1.053×10^{-5}	0.98431	0.98039	0.136665	0.165327	7	5
$Cu_{0.5}Co_{0.5}Fe_2O_4$	−0.495000	0.168111	**1.4690**	0.1253	1.1469–1.7911	11.724	7.937×10^{-5}	0.96490	0.95613	0.197829	0.198911	7	5

Note: Pseudo-first-order kinetic parameters were obtained from linear regression of $\ln(C_0/C_t)$ versus irradiation time over 30–210 min (n = 7, df = 5). k_1 corresponds to the regression slope. SE denotes the standard error of the estimated parameter; 95% CI denotes the two-sided 95% confidence interval calculated using the Student's t distribution. SSE and RMSE represent the residual sum of squares and root-mean-square error, respectively. The 240-min data point was excluded from regression fitting and used only for the final degradation efficiency. The pseudo-first-order model was employed as an empirical comparative descriptor rather than as a mechanistic assignment of reaction order.

Table S6. Comparison of zero-order, pseudo-first-order, and pseudo-second-order kinetic models.

Catalyst	Model	A ± SE	k (min^{-1})	SE(k)	95% CI of k	R^2	Adj. R^2	RMSE	SSE	AICc	ΔAICc	Akaike weight
**x=0**	Zero-order	0.8532 ± 0.0288	0.002958	0.000215	0.002406–0.003510	0.97431	0.96147	0.02881	0.005812	**−35.657**	**0.000**	**0.942**
	PFO	0.9374 ± 0.0642	0.005722	0.000702	0.003918–0.007525	0.94180	0.91270	0.04337	0.013167	−29.932	5.725	0.054
	PSO	1.0444 ± 0.1633	0.010102	0.002067	0.004789–0.015414	0.87709	0.81563	0.06303	0.027808	−24.698	10.958	0.004
**x=0.1**	Zero-order	0.9574 ± 0.0205	0.003523	0.000153	0.003130–0.003915	0.99069	0.98604	0.02049	0.002938	−40.431	0.579	0.428
	PFO	1.0948 ± 0.0308	0.006597	0.000304	0.005816–0.007379	**0.99143**	**0.98715**	**0.01966**	**0.002705**	**−41.010**	**0.000**	**0.571**
	PSO	1.3090 ± 0.1608	0.010974	0.001449	0.007250–0.014699	0.94645	0.91967	0.04914	0.016904	−28.183	12.828	0.001
**x=0.2**	Zero-order	0.9321 ± 0.0315	0.003979	0.000235	0.003375–0.004584	0.98285	0.97427	0.03154	0.006964	−34.390	2.765	0.200
	PFO	1.1477 ± 0.0469	0.008794	0.000503	0.007501–0.010088	**0.98844**	**0.98267**	**0.02589**	**0.004691**	**−37.156**	**0.000**	**0.798**
	PSO	1.5880 ± 0.3753	0.016189	0.002809	0.008969–0.023408	0.92624	0.88935	0.06541	0.029948	−24.179	12.976	0.001
**x=0.3**	Zero-order	1.0093 ± 0.0278	0.004341	0.000207	0.003808–0.004874	**0.98871**	**0.98307**	**0.02783**	**0.005422**	**−36.143**	**0.000**	**0.990**
	PFO	1.2124 ± 0.0964	0.008562	0.000965	0.006080–0.011043	0.95752	0.93627	0.05399	0.020407	−26.864	9.279	0.010
	PSO	1.6027 ± 0.4712	0.014467	0.003348	0.005861–0.023073	0.87710	0.81564	0.09184	0.059036	−19.429	16.714	0.0002
**x=0.4**	Zero-order	0.8969 ± 0.0774	0.004646	0.000577	0.003164–0.006129	0.92847	0.89270	0.07738	0.041911	−21.827	10.505	0.005
	PFO	1.3745 ± 0.0953	**0.014332**	0.001142	0.011398–0.017267	**0.98405**	**0.97608**	**0.03654**	**0.009345**	**−32.332**	**0.000**	**0.993**
	PSO	4.9772 ± 7.6088	0.030423	0.008027	0.009789–0.051057	0.89619	0.84428	0.09322	0.060826	−19.220	13.112	0.001
**x=0.5**	Zero-order	0.9243 ± 0.0410	**0.004439**	0.000305	0.003653–0.005224	**0.97686**	**0.96530**	**0.04099**	**0.011759**	**−30.723**	**0.000**	**0.790**
	PFO	1.2205 ± 0.1044	0.011069	0.001196	0.007994–0.014144	0.96615	0.94923	0.04957	0.017203	−28.060	2.663	0.209
	PSO	2.0323 ± 1.0849	0.021632	0.005741	0.006873–0.036391	0.87071	0.80606	0.09689	0.065717	−18.678	12.045	0.002

Table S7. Apparent rate constants (kapp) and physicochemical descriptors of $Cu_xCo_{1-x}Fe_2O_4$ at different Cu-substitution levels (x = 0–0.5), including crystallite size. (D), dislocation density (δ), lattice parameter (a), FTIR band positions (ν_1 and ν_2), and optical band-gap energy (Eg).

x	kapp ×10^{-2} min^{-1}	D (nm)	δ ×10^{3} nm^{-2}	a (Å)	ν_1 (cm^{-1})	ν_2 (cm^{-1})	Eg (eV)
0.0	0.62	26.53	1.421	8.386	551.64	424.34	1.60
0.1	0.69	24.54	1.661	8.408	547.78	453.27	1.34
0.2	0.98	24.41	1.679	8.387	547.70	474.49	1.36
0.3	1.05	16.79	3.548	8.424	547.78	474.49	1.40
0.4	1.84	16.36	3.736	8.408	555.58	422.41	1.44
0.5	1.49	25.14	1.582	8.408	553.57	453.27	1.50

Table S8. Spearman rank-correlation analysis between k_{app} and physicochemical descriptors of $Cu_xCo_{1-x}Fe_2O_4$.

**Descriptor**	**n**	**Spearman ρs**	**Exact two-sided PP**
Crystallite size, D (nm)	6	−0.657	0.175
Lattice parameter, a (Å)	6	+0.577	0.283
FTIR ν1_1 (cm^{-1})	6	+0.551	0.272
FTIR ν2 (cm^{-1})	6	−0.147	0.778
Optical band gap, Eg (eV)	6	+0.086	0.919

Note: n denotes the number of independent composition levels. Exact two-sided PP-values were obtained by permutation testing. None of the associations reached $P<0.05$. Correlation coefficients are descriptive and should not be interpreted as evidence of causality.

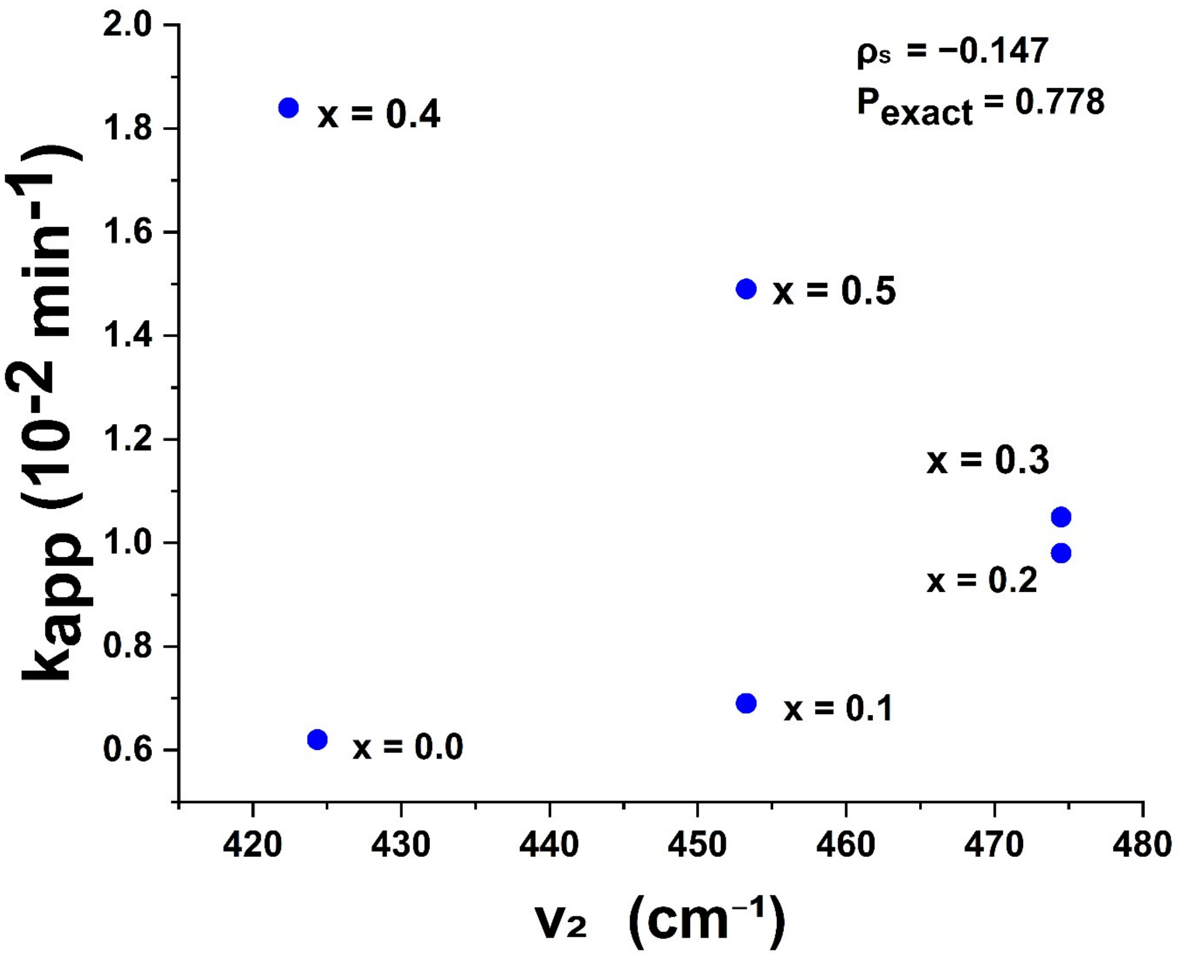

Fig. S1. Relationship between the FTIR ν₂ band position and the apparent rate constant (*k*app) for RhB degradation across the $Cu_xCo_{1-x}Fe_2O_4$ series. Each point represents one composition level (x = 0–0.5). The solid markers indicate the measured *k*app values, and the adjacent labels identify the corresponding Cu substitution level. The Spearman rank-correlation coefficient (ρ_s) and exact two-sided permutation *P*-value are shown in the panel. The ν_2–*k*app association was weak and statistically non-significant ($\rho_s = -0.147$, *P*exact = 0.778).

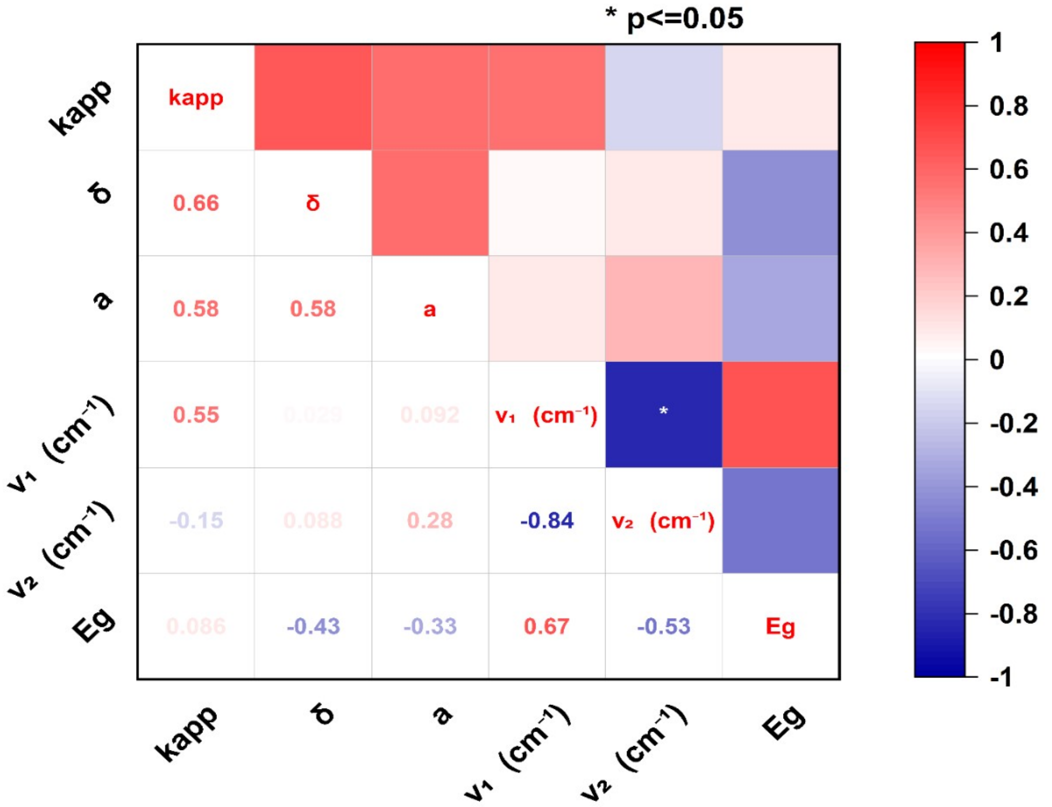

Fig. S2. Spearman rank-correlation matrix for ***k***app and the physicochemical descriptors of the $Cu_xCo_{1-x}Fe_2O_4$ series. The matrix summarizes pairwise Spearman rank-correlation coefficients (ρ_s) among *k*app, crystallite size (*D*), dislocation density (δ), lattice parameter (*a*), FTIR ν_1 and ν_2 band positions, and optical band-gap energy (*E*g). The colour scale represents the magnitude and direction of ρ_s, with red and blue denoting positive and negative associations, respectively. Asterisks indicate statistically significant two-sided correlations based on exact permutation tests ($P \leq 0.05$). Dislocation density (δ) was calculated directly from crystallite size ($\delta = 1/D^2$) and was therefore not treated as an independent descriptor in the activity-correlation analysis. The complete numerical correlation and significance results are provided in Table S8 (SI).
